# Sac7 and Rho1 regulate the white-to-opaque switching in *Candida albicans*

**DOI:** 10.1038/s41598-018-19246-9

**Published:** 2018-01-17

**Authors:** Siwy Ling Yang, Guisheng Zeng, Fong Yee Chan, Yan-Ming Wang, Dongliang Yang, Yue Wang

**Affiliations:** 1 Institute of Molecular and Cell Biology, 61 Biopolis Drive, Proteos, Singapore, 138673 Singapore; 2 Institute of Advanced Materials, Nanjing University of Posts and Telecommunications, Nanjing, Jiangsu 210046 People’s Republic of China; 3 Department of Biochemistry, Yong Loo Lin School of Medicine, National University of Singapore, Singapore, 117597 Singapore

## Abstract

*Candida albicans* cells homozygous at the mating-type locus stochastically undergo the white-to-opaque switching to become mating-competent. This switching is regulated by a core circuit of transcription factors organized through interlocking feedback loops around the master regulator Wor1. Although a range of distinct environmental cues is known to induce the switching, the pathways linking the external stimuli to the central control mechanism remains largely unknown. By screening a *C. albicans* haploid gene-deletion library, we found that *SAC7* encoding a GTPase-activating protein of Rho1 is required for the white-to-opaque switching. We demonstrate that Sac7 physically associates with Rho1-GTP and the constitutively active Rho1^G18V^ mutant impairs the white-to-opaque switching while the inactive Rho1^D124A^ mutant promotes it. Overexpressing *WOR1* in both *sac7Δ/Δ* and *rho1*^*G18V*^ cells suppresses the switching defect, indicating that the Sac7/Rho1 module acts upstream of Wor1. Furthermore, we provide evidence that Sac7/Rho1 functions in a pathway independent of the Ras/cAMP pathway which has previously been positioned upstream of Wor1. Taken together, we have discovered new regulators and a signaling pathway that regulate the white-to-opaque switching in the most prevalent human fungal pathogen *C. albicans*.

## Introduction

The fungus *Candida albicans* (*Ca*) is usually a harmless member of the human microbiota colonizing diverse host niches such as the oral cavity and gastrointestinal and urogenital tracts^1^. *C. albicans* is also an opportunistic pathogen responsible for millions of mucosal infections every year in otherwise healthy individuals and can cause life-threatening systemic infections in immunocompromised patients with high mortality rates^1,2^. The ability to switch between different morphological forms, such as yeast and hyphae, is thought to be a critical determinant of *C. albicans* virulence^1,3^.

Another well-studied morphological change seen in *C. albicans* is the white-to-opaque phenotypic transition^4,5^. White cells are oval-shaped and form white, hemispherical colonies with a smooth surface typical of the ones formed by standard *C. albicans* strains, while opaque cells are elongated and generate large, flat gray colonies^5,6^. Opaque cells often have a giant vacuole in the cytoplasm and possess pimple-like structures on the cell wall, and opaque colonies become pink/red when grown in the presence of Phloxine B (PB), a dye routinely used to differentiate opaque from white colonies^5–7^.

The biological role of the white-to-opaque transition is intimately related to mating^8,9^, a rare event in the life cycle of *C. albicans* but important for generating genetic diversity for adaption to changing environments^10^. Only opaque cells are capable of mating. The vast majority of natural *C. albicans* isolates are diploids and heterozygous at the mating-type locus (*MTL*a/α) which encodes an a1-α2 transcriptional co-repressor that keeps *C. albicans* cells in the white state^8,9^. To mate, the a/α white cells must first undergo homozygosis at the *MTL* to produce a/a or α/α cells, enabling the switch to the opaque state^9^. A low level of spontaneous white-to-opaque switching occurs and has been shown to be required for the formation of *MTL*-homozygous white cell “sexual” biofilms which facilitate the mating of minority opaque cells^11,12^. Moreover, this phenotypic transition may also assist host commensalism and pathogenesis^4^. For example, opaque cells have lost the capacity to release a potent chemoattractant for human polymorphonuclear leukocytes^13^, providing a possible mechanism for opaque cells to avoid phagocytosis^14^.

The transcription factor Wor1 is a master regulator of the white-to-opaque switching^15–17^. Deletion of *WOR1* locks the *MTL*-homozygous cells in the white phase, while overexpression of *WOR1* promotes the formation of opaque cells even in *MTL*-heterozygous strains^15–17^. Wor1, together with at least five other transcription factors (Czf1, Efg1, Wor2, Ahr1, and Wor3), form an interlocking network of activation and repression that governs the white and opaque phenotypes^18,19^. The components of this regulatory network are subject to regulation by several layers of controls^20^ such as changes in chromatin state through histone and DNA modification, the phosphorylation and dephosphorylation of key regulators, the configuration of the *MTL* locus, and the participation of the mediator complex^21–23^.

Environmental signals have a strong influence on the frequency of the white-to-opaque switching and the stability of the opaque phenotype. Some environmental cues can even override the exclusivity of switching in the *MTL*-homozygous background. These external signals are extremely diverse. For examples, low temperature (24 °C) promotes white-to-opaque switching while higher temperatures (30 °C and 37 °C) inhibit it^5^. Physiological levels of CO_2_ induce the white-to-opaque switching and stabilize the opaque phenotype^24^. Acidic pH increases the switching from white to opaque state under certain culture conditions^25^. When N-acetylglucosamine (GlcNAc) is used as the sole sugar source, it serves as a potent inducer of the white-to-opaque switching^26^. UV irradiation stimulated switching in both white-to-opaque and opaque-to-white directions^27^. Genotoxic stress generated by chemical treatment (MMS and HU) or gene deletion (*RAD51* and *RAD52*) and oxidative stress produced by hydrogen peroxide all induce efficient white-to-opaque switching^28^. A largely unanswered question is how these diverse environmental signals cause corresponding changes in the activity of the central transcription network to effect the white-to-opaque phenotypic transition. The Ras1/cAMP signaling cascade is a primary signal transduction pathway that mediates a range of cellular responses, including the white-to-opaque and yeast-hypha transition, to various environmental signals^26^. The adenylyl cyclase Cyr1, a large protein and an essential component of the Ras1/cAMP pathway, carried multiple signal sensors along its length^29^. However, the mechanisms that sense and transduce external signals to the central transcriptional control of the white-to-opaque switching is likely to be much more complex than what is currently known^4^.

Molecular genetics is a powerful tool for the discovery of mechanisms that control a biological process. However, standard genetic screens had largely been inapplicable in *C. albicans* because of its diploid genome. This situation has begun to improve with the recent discovery of haploid *C. albicans* and the construction of tool strains^30,31^. Although the haploids were generated from heterozygous diploids through concerted chromosome loss and hence have a different genetic background from their parents, they inherited the defining characteristics of standard diploid *C. albicans* including the yeast-hypha transition, the white-to-opaque switching, and chlamydospore formation^30^. Screening a small haploid gene deletion library has led to the discovery of new regulators of biofilm formation and polarized growth^32,33^. As mating normally occurs between haploid cells in most eukaryotes, we thought that the haploid *C. albicans* would be particularly suitable for uncovering new mechanisms that control this biological event.

Previously, we constructed a haploid *C. albicans* gene deletion library covering most uncharacterized GTPases and their regulators listed in *Candida* Genome Database^32^. A significant number of the genes are related with Rho GTPases which are members of the Ras superfamily of small GTP-binding proteins^34^. Rho GTPases are molecular switches, cycling between an active GTP-bound form and an inactive GDP-bound form. GDP/GTP exchange factors (GEFs) activate Rho GTPases by promoting the formation of Rho1-GTP^34,35^, while GTPase-activating proteins (GAPs) inactivate Rho GTPases by enhancing GTP hydrolysis^34,36^. Rho GTPases are often positioned at the top of signal transduction pathways and interact with multiple downstream effectors to orchestrate various cellular processes important for cellular morphogenesis such as cytoskeletal dynamics, gene transcription, cell division, polarity establishment and maintenance, and membrane trafficking. Several GTPases and their regulators have been reported to control the yeast-hypha transition, a trait critical for the virulence of *C. albicans*^37,38^.

In this study, we screened the same haploid mutant library to look for new regulators of the white-to-opaque transition. We found that deletion of *SAC7*, a Rho1 GAP, significantly blocked the white-to-opaque switching in both haploid and *MTL*-homozygous diploid cells. Consistent with its role as a Rho1 GAP, we detected physical interaction of Sac7 with GTP-Rho1 but not GDP-Rho1. Expression of a constitutively active Rho1 caused a switching defect similar to that caused by the *SAC7* deletion. Overexpression of *WOR1* rescued the switching defects in *SAC7* deletion mutants and the strain expressing the active Rho1, while deletion of *RAS1* exacerbated the switching defect of the *sac7Δ/Δ* mutant. Our results indicate that Sac7/Rho1 acts upstream of Wor1 and independent of the Ras1/cAMP pathway. Thus, our findings have identified Sac7 and Rho1 as key elements of a novel signaling pathway that regulates the white-to-opaque transition in *C. albicans*.

## Results

### Haploid *sac7Δ* mutant is defective in the white-to-opaque switching

Using the stable haploid strain GZY803, we constructed a gene deletion library of GTPases and their regulators^32^, most of which had not been characterized. To identify new regulators of the white-to-opaque switching, we screened the library for mutants unable to do so under an inducing condition (YPD, pH 6.0). Following a published protocol^25^, we spread haploid white cells on pH 6.0 YPD plates supplemented with PB and incubated the plates in the dark at 25 °C for 6–7 days. While nearly all wild-type (WT) colonies became pink, the majority of the colonies from 5 mutants (*sac7Δ*, *bem2Δ*, *trs120Δ*, *yip4Δ*, and *nug1Δ*) remained white, indicating defects in the white-to-opaque switching (Fig. S1). We chose *sac7Δ* for further analyses, because *SAC7* encodes a putative GTPase-activating protein for the important signaling molecule Rho1.

**Figure 1:**
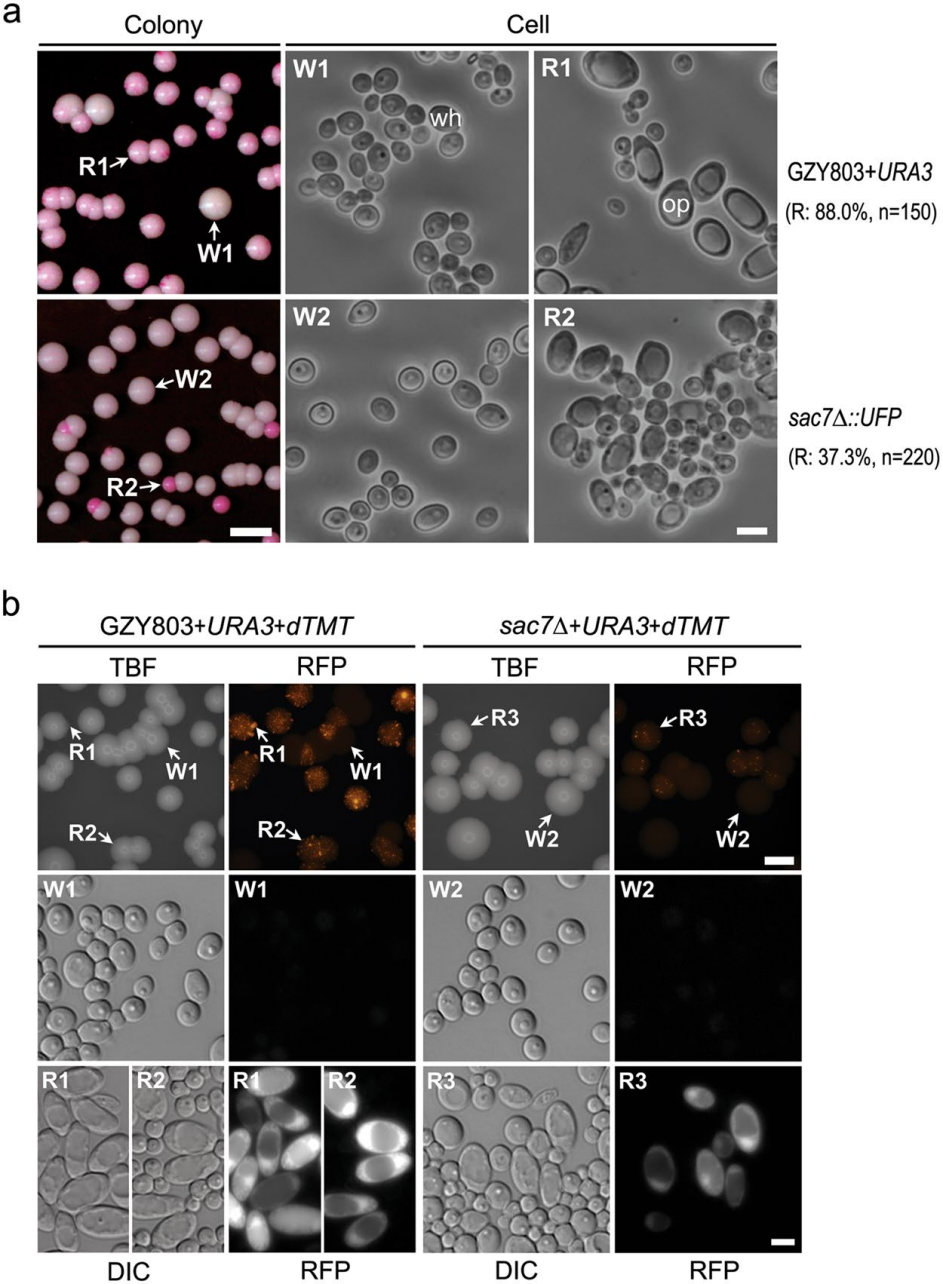
Haploid *sac7Δ* mutant is defective in the white-to-opaque switching. (**a**) Haploid WT GZY903 (GZY803 + *URA3*) and *sac7Δ* mutant (GZY916) cells were spread onto YPD plates (pH 6.0) containing 5 µg/ml PB and incubated at 25 °C in the dark for 7 days to allow the development of single colonies. Cells from representative colonies (W1 and R1) were picked and suspended into water for morphological examination under a microscope. n indicates the number of colonies analyzed in each strain. (**b**) Cells of GZY1121 (GZY803 + *URA3* + *dTMT*) and GZY1141 (*sac7Δ* + *URA3* + *dTMT*) were spread onto YPD plates (pH 6.0, +PB) and incubated at 25 °C in the dark for 7 days. The plates were examined under a fluorescent microscope to visualize colonies through the transmitted bright field (TBF) channel and detect dTomato signals through the RFP channel (same exposure time for the strains). Cells from various colonies (W1, W2 and R1 to R3) were picked and suspended into water for morphological examination (DIC) and dTomato signal detection (RFP) under a fluorescent microscope. Throughout this paper, bars on colony plates and cell images represent 5 mm and 5 µm, respectively.

Under the inducing condition, 88.0% (n = 150) of the colonies of the WT haploid GZY903 (GZY803 + *URA3*) cells were stained pink, some with red dots or sectors, while the rest were white (Fig. 1a). Microscopic examination revealed that the white colonies contained only white cells (wh) which were round or ellipsoidal (Fig. 1a, W1). In contrast, the majority of cells from the pink/red colonies exhibited characteristics of typical opaque cells (op) which were large and elongated with a huge cytoplasmic vacuole (Fig. 1a, R1). Under the same inducing condition, only 37.3% (n = 220) of the *sac7Δ* colonies were pink/red (Fig. 1a). Cells from white *sac7Δ* colonies were all white cells (Fig. 1a, W2) while those in the pink/red colonies were a mixture of white and opaque cells (Fig. 1a, R2). The reduced number of PB-stained colonies of the *sac7Δ* mutant suggests that Sac7 may play a role in the white-to-opaque switching.

As we had noticed that cells of some *C. albicans* strains were stained pink by PB even under non-inducing condition (YPD, pH 7.0) and the cells had a typical yeast morphology, we sought to find a more reliable way to distinguish opaque and white cells. We used the promoter of the opaque-specific gene *OP4* to control the expression of *C. albicans* codon-optimized dTomato (dTMT)^39^, an exceptionally bright red fluorescent protein ideal for live cell imaging^40^. We introduced this construct into both GZY903 (WT) and the *sac7Δ* mutant to generate GZY803 + *URA3* + *dTMT* and *sac7Δ* + *URA3* + *dTMT* strains, respectively, and grew them under the inducing condition. When examined under a fluorescence microscope, the majority of the GZY803 + *URA3* + *dTMT* colonies strongly expressed dTomato as bright sectors or punctate dots, and only a few colonies/sectors lacked detectable signal and remained evenly dim (Fig. 1b). Further examination of GZY803 + *URA3* + *dTMT* colonies revealed that cells from the dim colonies were typical white cells without detectable dTomato signals (Fig. 1b, W1). In contrast, the majority of cells from the bright sectors were characteristic of opaque cells emitting strong red fluorescence (Fig. 1b, R1); and cells from the colonies with punctate dots were a mixture of white and opaque cells (Fig. 1b, R2). Therefore, expression of dTomato from the *OP4* promoter allows us to distinguish opaque from white cells not only on the colony level but also the cellular level. Under the same inducing condition, fewer colonies of *sac7Δ* + *URA3* + *dTMT* expressed dTomato and the signals were much weaker overall (Fig. 1b, W2 and R3), confirming that the *sac7Δ* mutant is defective in the white-to-opaque switching.

### The white-to-opaque switching defect of *sac7Δ* was rescued by *SAC7* and suppressed by *WOR1* overexpression

**Figure 2:**
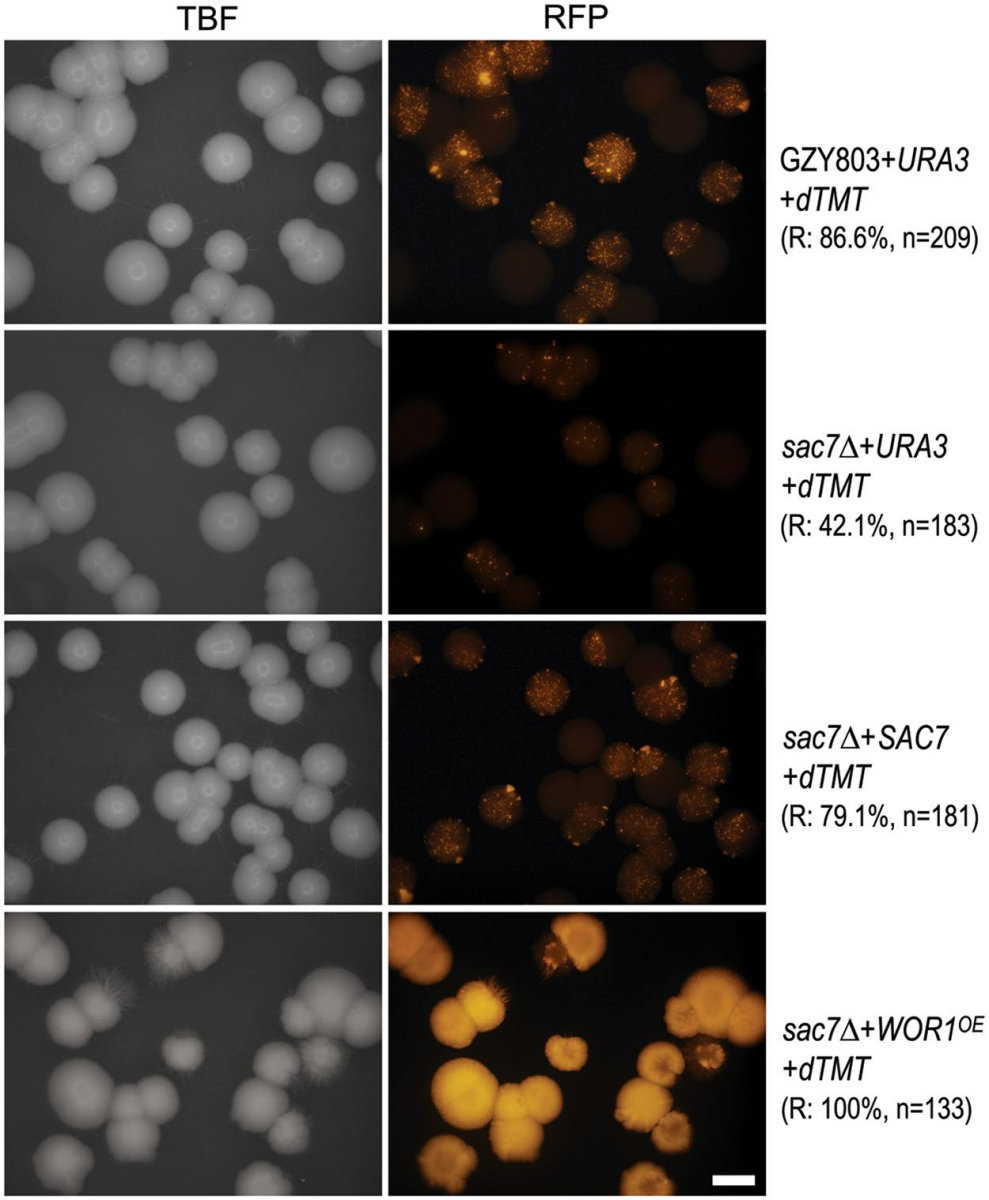
The defective white-to-opaque switching of *sac7Δ* is rescued by *SAC7* reintegration and suppressed by *WOR1* overexpression. Cells of GZY1121 (GZY803 + *URA3* + *dTMT*), GZY1141 (*sac7Δ* + *URA3* + *dTMT*), GZY1153 (*sac7Δ* + *SAC7* + *dTMT*), and GZY1130 (*sac7Δ* + *WOR1*^*OE*^ + *dTMT*) were spread onto YPD plates (pH 6.0, +PB) and incubated at 25 °C in the dark for 7 days. The plates were examined under a fluorescent microscope to capture colony images through the TBF channel and detect dTomato signals through the RFP channel with the same exposure time. The percentage of dTomato-expressing colonies of each strain was calculated.

To confirm that the loss of *SAC7* was indeed responsible for the white-to-opaque switching defect in the haploid *sac7Δ* mutant, we re-introduced a WT *SAC7* gene to generate a rescued strain *sac7Δ* + *SAC7* + *dTMT*. After the induction, 86.6% (n = 209) of WT haploid (GZY803 + *URA3* + *dTMT*) colonies produced strong dTomato signals (Fig. 2) while only 42.1% (n = 183) of *sac7Δ* mutant (*sac7Δ* + *URA3* + *dTMT*) colonies exhibited detectable but much weaker dTomato signals (Fig. 2). As expected, the rescued strain (*sac7Δ* + *SAC7* + *dTMT*) produced not only a significantly higher percentage of dTomato-expressing colonies (79.1%, n = 181) but also markedly enhanced fluorescence intensity to a level comparable to that of WT haploid colonies (Fig. 2). The results confirmed that the switching defect observed in *sac7Δ* was caused by the loss of *SAC7* but not other mutations.

Wor1 is the master regulator of the white-to-opaque switching^15^. While deletion of *WOR1* blocks the formation of opaque cells, overexpression of *WOR1* converts all cells to opaque cells even in *MTL*-heterozygous cells^15–17^. To investigate the epistatic relationship between *SAC7* and *WOR1*, we generated a strain that overexpressed *WOR1* (*WOR1*^*OE*^) from the tetracycline-suppressible (Tet-off) promoter in the *sac7Δ* background. Under the inducing condition, all colonies (n = 133) of the *sac7Δ* + *WOR1*^*OE*^ + *dTMT* strain produced strong dTomato signals (Fig. 2), indicating the suppression of the switching defect of the *sac7Δ* mutant by *WOR1* overexpression. The data, therefore, indicate that Sac7 functions upstream of Wor1 in the regulation of the white-to-opaque transition.

### Diploid *sac7Δ/Δ* mutant was also defective in the white-to-opaque switching

**Figure 3:**
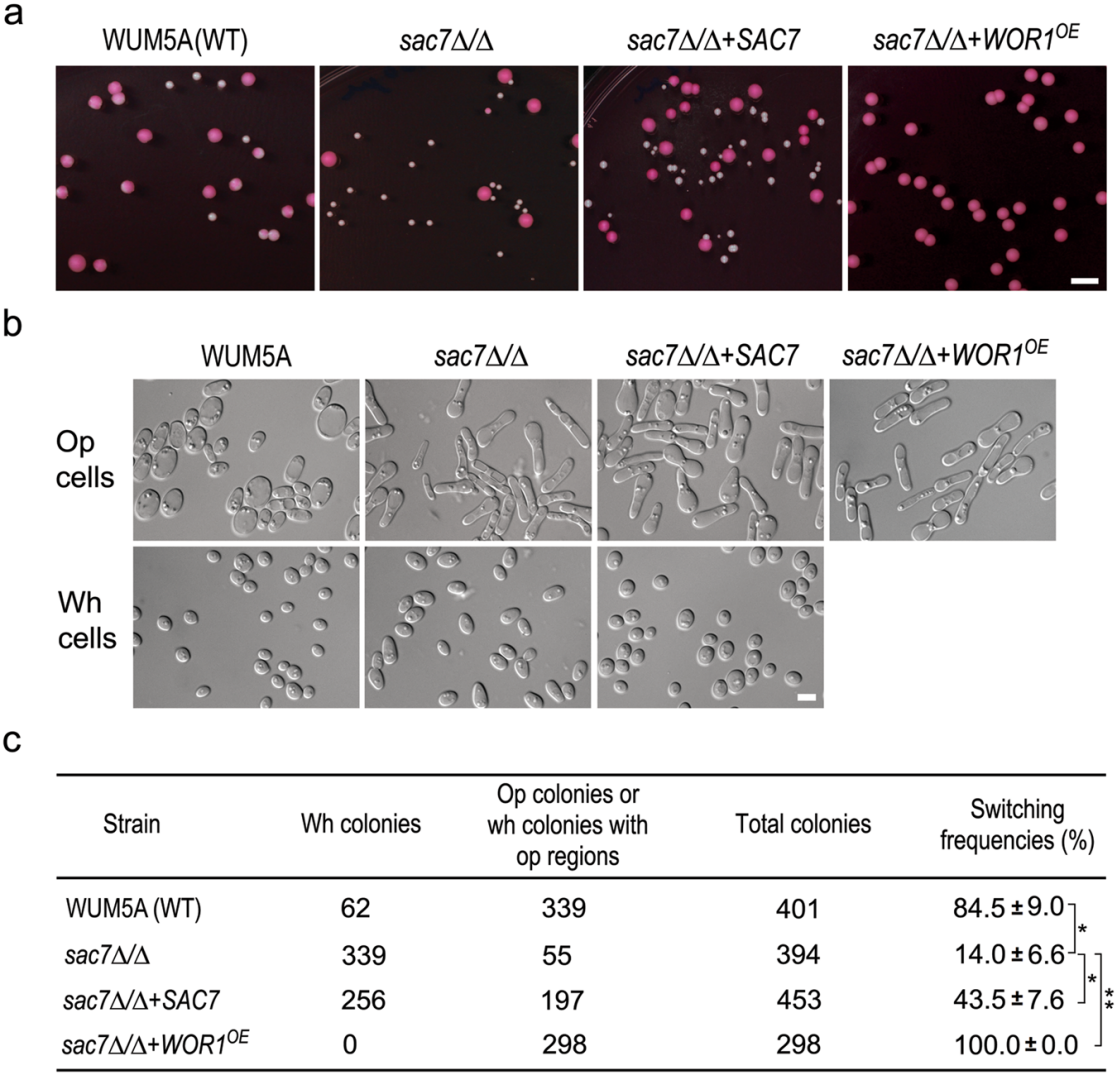
The diploid *sac7Δ/Δ* mutant is defective in the white-to-opaque switching. (**a**) Diploid cells of WUM5A (WT), *sac7Δ/Δ* (YSL504), *sac7Δ/Δ* + *SAC7* (YSL532), and *sac7Δ/Δ* + *WOR1*^*OE*^ (YSL505) were spread onto Lee’s GlcNAc plates (pH 6.0, +PB) and incubated at 25 °C in the dark for 6 days before photography. (**b**) Cells from randomly selected red and white colonies as indicated were suspended in water and examined under a microscope. (**c**) The white-to-opaque switching frequency of the strains grown on Lee’s GlcNAc plates (pH 6.0, +PB). White colonies, red colonies and white colonies with red regions from each strain were counted respectively, and the switching frequency was calculated as the percentage of red colonies plus white colonies with red regions over total colonies. *p < 0.05; **p < 0.01 (Student’s *t*-test).

To examine whether *SAC7* is also involved in the white-to-opaque switching in diploid cells, we generated a *sac7Δ/Δ* mutant in WUM5A^41^, an *MTL*-homozygous strain derived from WO-1 and routinely used in the studies of the white-to-opaque switching. To induce the switching, the diploid cells were first grown on GMM plates at 25 °C for 3 days and then collected in water before plating onto Lee’s GlcNAc plates (pH 6.0, +PB) for incubation in the dark at 25 °C for 6 days. Under this inducing condition, the majority of the WUM5A colonies were entirely or partly stained red by PB with only a few white colonies (Fig. 3a). Morphological examination confirmed that cells from the red colonies were typical opaque cells with pimples on cell surface while those in white colonies were white cells with smooth cell surface (Figs 3b and S2). WUM5A had a switching frequency of 84.5 ± 9.0% (Fig. 3c) while the switching frequency of the *sac7Δ/Δ* mutant was 14.0 ± 6.6% (Fig. 3a–c), indicating that the deletion of *SAC7* in diploid *C. albicans* cells severely impaired the white-to-opaque switching. Re-introduction of a WT *SAC7* gene back into the *sac7Δ/Δ* mutant increased the switching frequency to 43.5 ± 7.6% (Fig. 3a–c). Furthermore, overexpressing *WOR1* in *sac7Δ/Δ* cells (*sac7Δ/Δ* + *WOR1*^*OE*^) from the Tet-off promoter completely suppressed the white-to-opaque switching defect. All the colonies of the *sac7Δ/Δ* + *WOR1*^*OE*^ strain were stained red and contained only opaque cells (Fig. 3a–c). Similarly, the *sac7Δ/Δ* mutant was also defective in CO_2_-induced white-to-opaque switching, which could be largely rescued by re-introduction of a WT *SAC7* gene (Fig. S3). The results provide further evidence that Sac7 has a role in the regulation of the white-to-opaque switching which is mediated by Wor1 in both haploid and diploid cells.

### Overexpression of *SAC7* enhances the white-to-opaque switching

**Figure 4:**
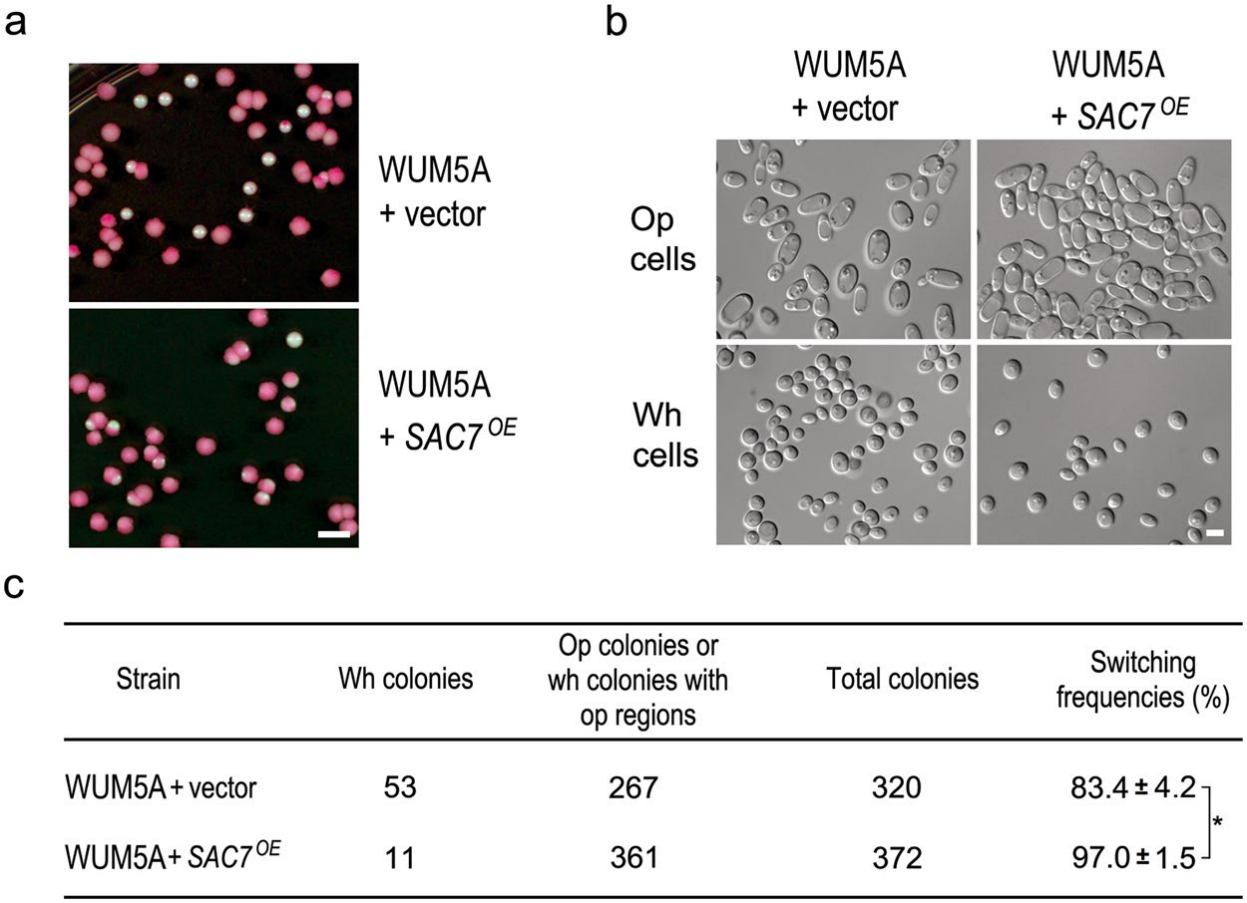
*SAC7* overexpression enhances the white-to-opaque switching. (**a**) Representative images of colonies formed by diploid cells of WUM5A + vector (YSL509) and WUM5A + *SAC7*^*OE*^ (YSL512) after incubation on Lee’s GlcNAc plates (pH 6.0, +PB) at 25 °C in the dark for 6 days. (**b**) Representative images of white and opaque cells formed by WUM5A + vector and WUM5A + *SAC7*^*OE*^ cells. (**c**) The white-to-opaque switching frequency for WUM5A + vector and WUM5A + *SAC7*^*OE*^ strains tested on Lee’s GlcNAc plates (pH 6.0, +PB). *p < 0.05 (Student’s *t*-test).

Since the deletion of *SAC7* reduced the white-to-opaque switching frequency, we expected that overexpression of *SAC7* would promote the switching. To test this idea, a *SAC7*-overexpressing plasmid driven by the Tet-off promoter were introduced into WUM5A (WUM5A + *SAC7*^*OE*^) and the switching frequency were determined on Lee’s GlcNAc plates (pH 6.0, +PB). After the induction, the WUM5A + *SAC7*^*OE*^ strain exhibited a switching frequency of 97.0 ± 1.5% which was significantly higher than the 83.4 ± 4.7% shown by the strain transformed with the empty vector (Fig. 4a,b,c). The data above indicate that Sac7 is a positive regulator of the white-to-opaque switching.

### Sac7 physically interacts with Rho1

**Figure 5:**
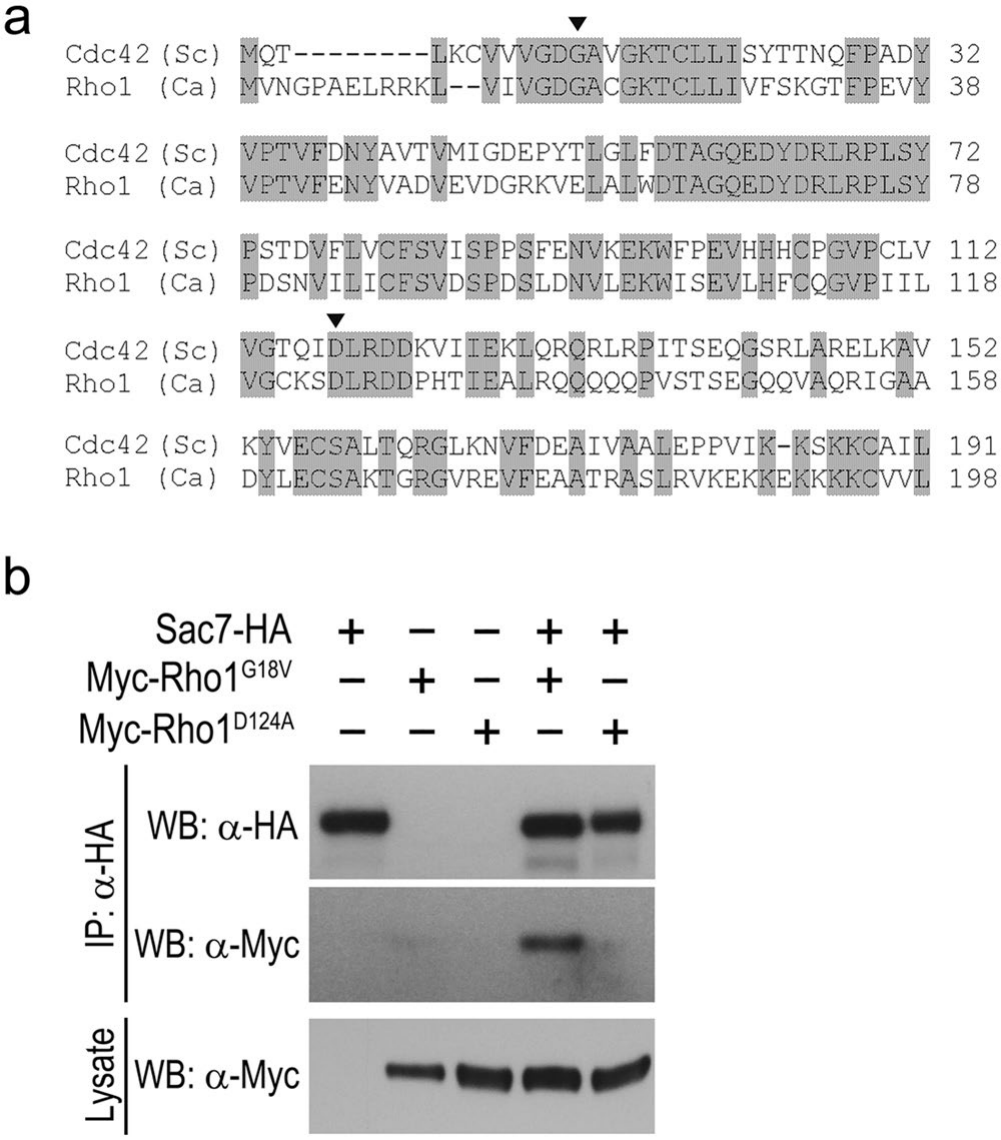
Sac7 physically interacts with Rho1. (**a**) Amino acid sequence alignment of *Ca*Rho1 with *Sc*Cdc42. Consensus residues are shaded. The arrowhead and asterisk indicate the conserved residues mutated to generate GTP- and GDP-locked forms of GTPase, respectively. (**b**) Sac7-HA interacts with Myc-Rho1^G18V^ but not Myc-Rho1^D124A^. Lysates prepared from cells expressing Sac7-HA (YSL607), Myc-Rho1^G18V^ (YSL570), Myc-Rho1^D124A^ (YSL564), Sac7-HA and Myc-Rho1^G18V^ (YSL601), and Sac7-HA and Myc-Rho1^D124A^ (YSL609) were immunoprecipitated with the HA antibody. The immunoprecipitates were separated by SDS-PAGE and transferred to PVDF membrane for WB analysis with HA and Myc antibodies. The cell lysates were also directly subjected to WB analysis with the Myc antibody as a control.

In *Saccharomyces cerevisiae* (*Sc*), Sac7 is known to be a GAP of Rho1 and physical interaction between them has been demonstrated. Next, we performed co-immunoprecipitation to determine whether Sac7 also interacts with Rho1 in *C. albicans*. Being aware of the cycling of Rho1 between active and inactive forms, we sought to construct constitutively active and inactive versions of Rho1. Sequence alignment of *Ca*Rho1 with the corresponding mutants of the Rho GTPase *Sc*Cdc42^42^ (Fig. 5a) guided us to generate two *C. albicans* strains expressing the Myc-Rho1^G18V^ (GTP-locked) and Myc-Rho1^D124A^ (GDP-locked) mutant GTPase, respectively. We constructed strains co-expressing Sac7-HA with Myc-Rho1^G18V^ or Myc-Rho1^D124A^, immunoprecipitated Sac7-HA with a HA antibody from cell extracts, and probed the precipitation products using anti-Myc WB. We could only detect the interaction of Sac7 with Myc-Rho1^G18V^ but not Myc-Rho1^D124A^ (Fig. 5b). As the negative control, in cell extracts expressing Myc-Rho1^G18V^ alone, we did not detect Rho1 in the anti-HA precipitation products, indicating that the detection of Myc-Rho1^G18V^ was due to specific interaction with Sac7-HA (Fig. 5b). Together, the results demonstrate that Sac7 physically associates with the active, GTP-bound form of Rho1 *in vivo*.

### Rho1-GTP negatively regulates the white-to-opaque switching

**Figure 6:**
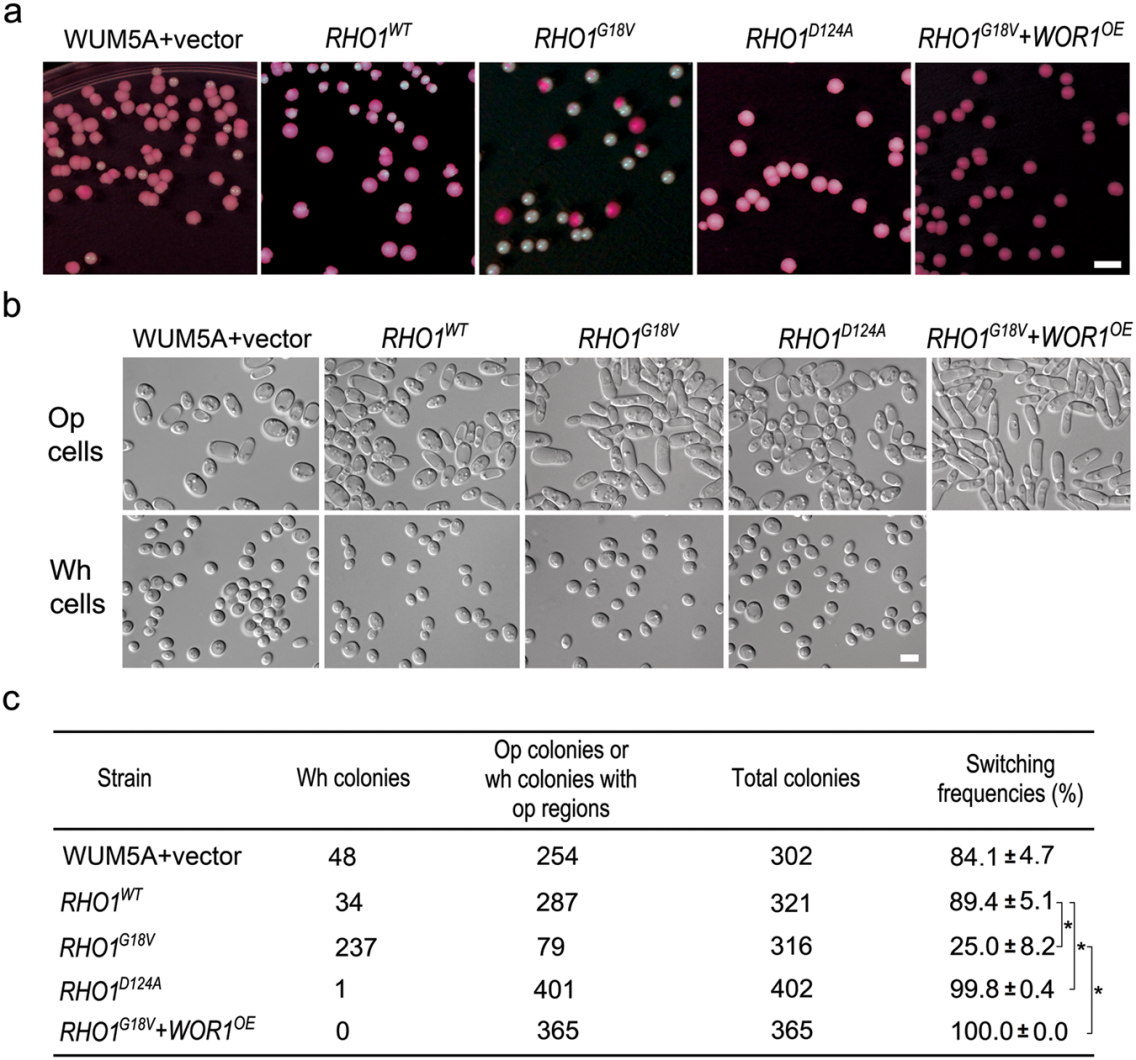
Rho1 negatively regulates the white-to-opaque switching. (**a**) Cells of WUM5A transformed with vector (YSL509), *RHO1*^*WT*^ (YSL617), *RHO1*^*G18V*^ (YSL608), *RHO1*^*D124A*^ (YSL613), or *RHO1*^*G18V*^ + *WOR1*^*OE*^ (YSL616) were grown on Lee’s GlcNAc plates (pH 6.0, +PB) at 25 °C in the dark for 6 days. Colonies formed by each strain were photographed and representative images are shown. (**b**) Representative images of white and opaque cells from the indicated strains. (**c**) The white-to-opaque switching frequency for WUM5A cells transformed with vector, *RHO1*^*WT*^, *RHO1*^*G18V*^, *RHO1*^*D124A*^, or *RHO1*^*G18V*^ + *WOR1*^*OE*^. *p < 0.05 (Student’s *t*-test).

Above, we have provided evidence that Sac7 promotes the white-to-opaque transition. As Sac7 is a GAP of Rho1, we hypothesized that Rho1-GTP would negatively regulate the white-to-opaque switching. To test this hypothesis, we overexpressed, from the Tet-off promoter, the WT (*RHO1*^*WT*^), constitutively active (*RHO1*^*G18V*^), or constitutively inactive (*RHO1*^*D124A*^) *RHO1* in WUM5A. The white-to-opaque switching frequency was determined by growing cells on Lee’s GlcNAc plates (pH 6.0, +PB) in the dark at 25 °C for 6 days. The results showed that the switching frequencies of cells overexpressing *RHO1*^*WT*^, *RHO1*^*G18V*^ and *RHO1*^*D124A*^ were 89.4 ± 5.1%, 25.0 ± 8.2% and 99.8 ± 0.4%, respectively (Fig. 6a–c). A similar pattern was observed when these strains were subjected to CO_2_-induced white-to-opaque switching assay (Fig. S4). The data demonstrate that the active Rho1-GTP negatively regulates the white-to-opaque switching, which is consistent with the effect of *SAC7* deletion and overexpression on the switching.

To investigate whether the regulation of the white-to-opaque switching by Rho1 is also mediated by Wor1, we overexpressed *WOR1* in *RHO1*^*G18V*^ cells. The switching assay showed that all the colonies of *RHO1*^*G18V*^ + *WOR1*^*OE*^ cells were stained red and contained only opaque cells (Fig. 6a,b) in sharp contrast to the 25.0 ± 8.2% switching frequency shown by the *RHO1*^*G18V*^ cells (Fig. 6c). The data indicate that Rho1 functions upstream of Wor1.

### *ras1Δ/Δ* exacerbates the white-to-opaque switching defect of *sac7Δ/Δ* mutant

**Figure 7:**
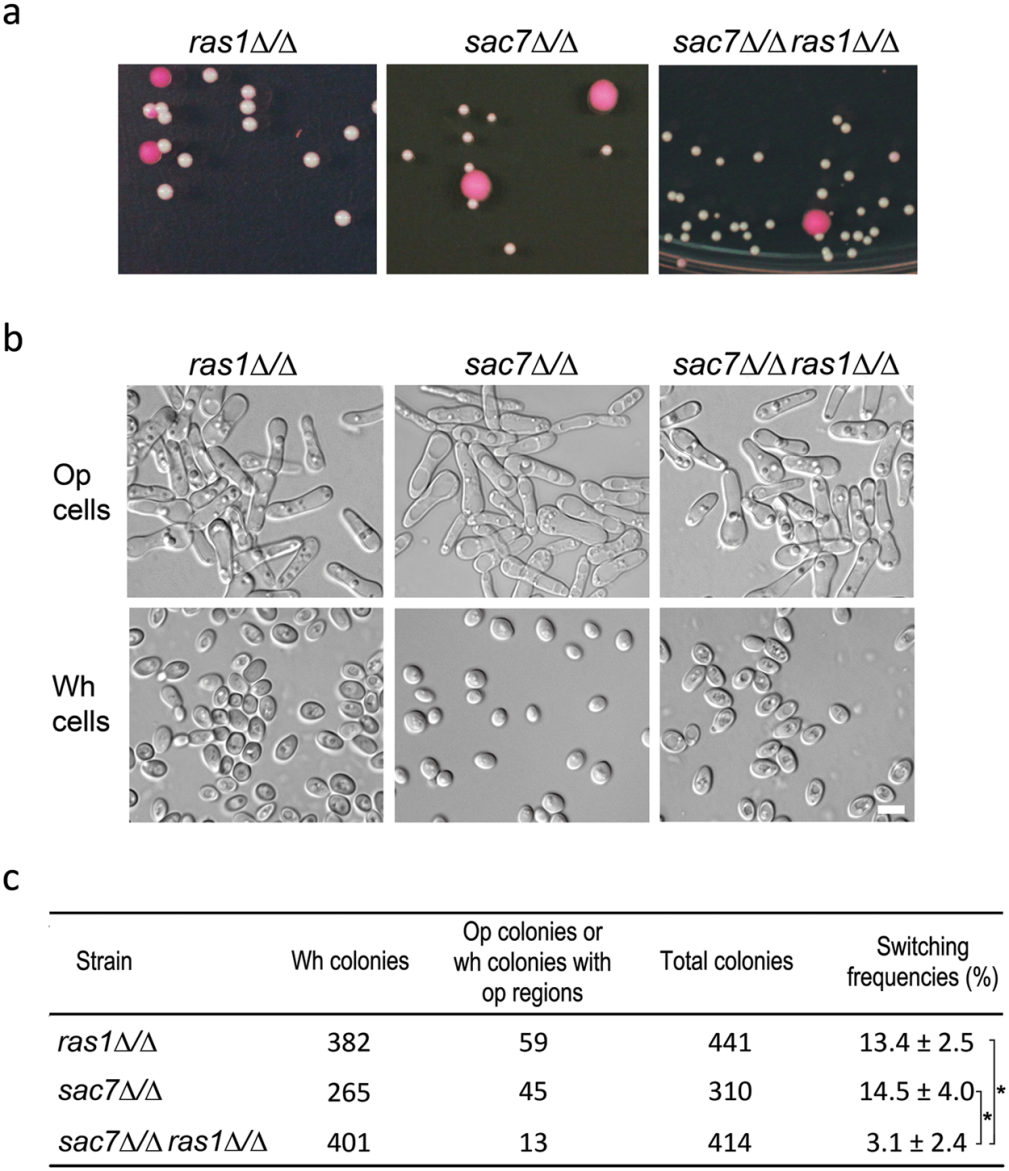
The white-to-opaque switching defect of the *sac7Δ/Δ* mutant was further exacerbated by *ras1Δ/Δ*. (**a**) Cells of *ras1Δ/Δ* (YSL639), *sac7Δ/Δ* (YSL503), and *sac7Δ/Δ ras1Δ/Δ* (YSL623) were grown on Lee’s GlcNAc plates (pH 6.0, +PB) at 25 °C in the dark for 6 days before photography. (**b**) Representative images of white and opaque cells of the indicated strains. (**c**) The white-to-opaque switching frequency of the tested strains. *p < 0.05 (Student’s *t*-test).

A previous study has shown that the Ras1/cAMP signaling pathway plays an important role in the white-to-opaque switching of *C. albicans* and acts upstream of Wor1^26^. To explore the relationship between Rho1/Sac7 and the Ras1/cAMP pathway, we constructed a *sac7Δ/Δ ras1Δ/Δ* double mutant and tested its ability to undergo the white-to-opaque switching. The *sac7Δ/Δ ras1Δ/Δ* mutant displayed a significantly lower switching frequency than either one of the single mutants deleted of *SAC7* or *RAS1* alone (Fig. 7a,b). The switching frequencies were 3.1 ± 2.4% for *sac7Δ/Δ ras1Δ/Δ*, 14.5 ± 4.0% for *sac7Δ/Δ*, and 13.4 ± 2.5% for *ras1Δ/Δ* cells (Fig. 7c). The results suggest that Rho1/Sac7 function in a pathway in parallel with the Ras1/cAMP signaling cascade to regulate the white-to-opaque switching in *C. albicans*.

## Discussion

In this study, we have identified Sac7 as a new regulator of the white-to-opaque switching in *C. albicans*. Sac7 is a GAP that negatively regulates the small GTPase Rho1. Our data indicate that Sac7 promotes the white-to-opaque switching by converting Rho1 from its GTP-bound active to GDP-bound inactive form. Epistatic analyses revealed that Rho1/Sac7 functions in a signaling pathway in parallel with the Ras1/cAMP cascade to regulate the phenotypic switching. Hence, our study has discovered a new signaling pathway that controls a unique and important trait in the life cycle of the fungal pathogen *C. albicans*.

Before this study, the function of *C. albicans* Sac7 was uncharacterized. Based on its closest homologue in *S. cerevisiae*, the protein is inferred to act as a Rho1 GAP. Our experiments have provided compelling evidence supporting this role. By co-immunoprecipitation, we demonstrated the physical association of Sac7 with the active Rho1^G18V^ but not the inactive Rho1^D124A^ protein *in vivo* (Fig. 5b). Also, the strain overexpressing *SAC7*, which drives Rho1 towards the Rho1-GDP form, exhibited an increased white-to-opaque switching frequency to a level similar to that of the constitutively inactive Rho1^D124A^ mutant (Figs 4 and 6). Also, the *sac7Δ/Δ* mutant, in which Rho1 is kept in the active Rho1-GTP form, showed significantly reduced switching frequencies like the constitutively active Rho1^G18V^ mutant (Figs 3 and 6). In *S. cerevisiae*, Sac7 plays an important role in the organization of actin cytoskeleton^43,44^ and cell wall organization^45,46^. We have observed that the *sac7Δ/Δ* diploid mutant of *C. albicans* was sensitive to several cell wall toxins including SDS, Calcofluor White, and Congo Red (Fig. S5), consistent with a role for Sac7 in maintaining the cell wall integrity.

In *S. cerevisiae*, Rho1 is an important regulator of diverse cellular functions including cell polarity, actin cytoskeleton organization, and cell wall biosynthesis^47^. It executes multiple tasks by binding and activating different effectors, such as Fks1 for cell wall remodeling^48–50^, Pkc1 for cell integrity signaling^50–52^, Bem4 for bud emergence^53^, Bni1 for actin cytoskeleton reorganization^54^, and Sec3 for exocytosis^55^. In *C. albicans*, Rho1 has been shown to interact directly with Gsc1 (also known as Fks1), the β-1,3-glucan synthase catalytic subunit^56^ and regulates cell wall biosynthesis. Depletion of Rho1 resulted in cell death, lysis, and aggregation and the failure to colonize the kidney of mice during systemic infection^57^, indicating an essential role for Rho1 in cell viability and virulence. Rho1 is also required for *C. albicans* invasive filamentous growth^38^. Recently, Rho1 was found to regulate morphogenesis via interaction with the GAP Lrg1^58^. In addition to cell wall integrity and morphogenesis, whether Rho1 also regulates other cellular processes in *C. albicans* remains to be determined. Our study expands Rho1’s role to the regulation of the white-to-opaque switching in *C. albicans*.

**Figure 8:**
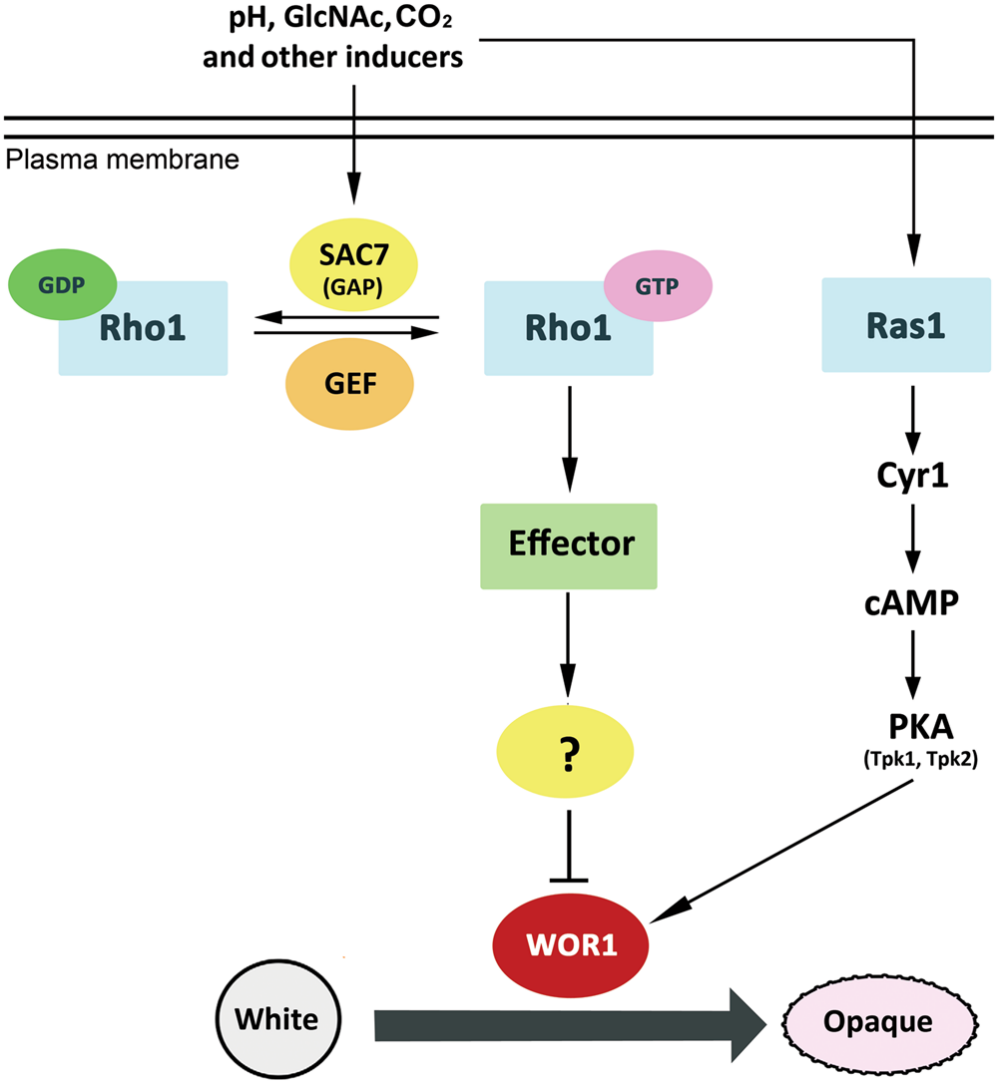
A schematic illustration of the role of Sac7/Rho1 in the regulation of the white-to-opaque switching (see the text for explanation).

A diverse range of environmental signals is known to influence the white-to-opaque switching in *C. albicans*^4^. However, our knowledge of the signaling pathways linking the external signals to the circuit of transcription factors that directly control the switching remains limited. The Ras1/cAMP pathway has been demonstrated to mediate the white-to-opaque switching in response to GlcNAc and CO_2_^26^. Our finding that *sac7Δ* and *ras1Δ* act synergistically to repress the switching (Fig. 7) suggests that Sac7/Rho1 and Ras1 function in independent pathways. On the other hand, the suppression of the white-to-opaque switching deficiency of *sac7Δ/Δ* and *rho1*^*G18V*^ mutants by *WOR1* overexpression (Figs 3 and 6) indicates that, like the Ras/cAMP pathway, Sac7/Rho1 functions upstream of Wor1. Here, we propose a working model to illustrate how Sac7/Rho1 regulates the white-to-opaque switching (Fig. 8). Under non-inducing conditions, Sac7 is inactive and Rho1 is thus in the active Rho1-GTP form. The active Rho1 then binds and activates its effector(s) which in turn activates downstream targets, leading to the inhibition of Wor1 and the white-to-opaque switching. Under inducing conditions, Sac7 is activated and Rho1-GTP is converted to the inactive Rho1-GDP form. Consequently, the Rho1 effector(s) becomes inactive and the inhibition on Wor1 is relieved, allowing cells to undergo the white-to-opaque switching. Currently, the identity of the effector(s) of Rho1 and the downstream signaling pathways linking to Wor1 remain unclear. One possibility is that Rho1, like its counterpart in *S. cerevisiae*, may interact with Pkc1 to initiate the mitogen-activated protein (MAP) kinase cascade^59^, eventually leading to the phosphorylation and activation of the MAP kinase Mkc1 (the last member of the MAP cascade) which in turn directly or indirectly inhibits Wor1. In support of this model, we have observed that Mkc1 is hyper-phosphorylated in the *sac7Δ/Δ* mutant and *MKK2* (MAP kinase kinase) overexpressing cells. Furthermore, the white-to-opaque switching frequency of *MKK2* overexpressing cells was reduced to a level similar to that of *sac7Δ/Δ* (Fig. S6). However, the physical interaction between Rho1 and Pkc1 has not been demonstrated and whether Wor1 is phospho-regulated by Mkc1 remains to be determined, although Wor1 has been shown to be phosphorylated by protein kinase A (PKA)^26^. Further investigations are required to conclude whether the Mkc1 MAP kinase cascade connects Rho1/Sac7 to Wor1 in the regulation of the white-to-opaque switching.

In this study, we have only tested three inducers lower pH, GlcNAc and CO_2_ that promote the white-to-opaque switching via the Rho1/Sac7 signaling pathway. Many environmental cues, such as the sugar source, temperature, low doses of UV irradiation, genotoxic and oxidative stress, can influence the white-to-opaque switching and the stability of the opaque phenotype^4^. Whether Sac7/Rho1 also has a role in mediating cellular responses to these signals remains to be determined.

We noticed that the white and opaque colonies formed by haploids and diploids have some differences. For example, haploid opaque colonies are slightly smaller than white colonies (Figs 1 and 2). In contrast, diploid opaque colonies are much bigger than white colonies (Fig. 3a). The reason causing such differences is unclear, but it is worthy to point out that haploids and diploids were induced to form opaque colonies by different inducers. Haploids were induced on YPD (pH 6.0) plates, while diploids were induced on Lee’s GlcNAc plates (pH 6.0). We have tried to induce haploid cells on Lee’s GlcNAc plates, but only tiny colonies could be formed and they were too small for accurate counting of white and opaque colonies. We also noticed that the diploid opaque cells have two types of morphology: oval-shaped or bean-shaped. The opaque cells of the wild-type diploid strain (WUM5A) were oval-shaped while opaque cells of strains derived from the wild-type strain are either oval- or bean-shaped. For examples, the opaque cells of *sac7Δ/Δ* and *RHO1*^*G18V*^ strains are bean-shaped, whereas the opaque cells of *SAC7*^*OE*^ and *RHO1*^*D124A*^ strains are oval-shaped. Interestingly, the opaque cells of WUM5A changed their morphology from oval-shaped to bean-shaped after cultured in liquid Lee’s GlcNAc medium for three more days (Figs 3b and S2), suggesting that culture condition may play a role in determining the morphology of opaque cells. Although what causes the morphological change remains to be determined, it is evident that both oval- and bean-shaped cells were bona fide opaque cells, because they possessed the characteristics of opaque cells^5,6^, including bigger sizes, a pimpled surface, giant vacuoles, and staining by Phloxine B.

We have developed a more precise method than PB staining to distinguish opaque and white cells. We used the *OP4* promoter to control the expression of *C. albicans* codon-optimized red florescent protein dTomato^39^. As *OP4* is opaque-specific, the dTomato signal can only be detected in opaque cells. On the colony level, two types of dTomato signals could be observed. One is bright sectors containing only opaque cells, and the other has areas with punctate signals that contain both opaque and white cells. On the cellular level, the dTomato signal is distributed throughout the entire cytoplasm in opaque cells except for the vacuoles (Figs 1b and 2). In contrast, PB staining can only distinguish opaque cells from white cells on the colony level but not the cellular level. We found that PB stained not only opaque but also white colonies of diploid and haploid strains derived from SC5314 with ura3 auxotrophy, such as CAI4, BWP17 and GZY803, often leading to misidentification of opaque colonies. In comparison, dTomato driven by the *OP4* promoter offers a convenient and reliable method to distinguish opaque and white cells.

*C. albicans* haploids were discovered recently to be derived from diploid parents via the concerted loss of one set of chromosomes^30^. Although the haploids did not show virulence in the mouse model of systemic infection, they retained key characteristics that define the species, including the yeast-to-hyphae transition, the white-to-opaque switching, mating, and the formation of chlamydospores^30^. The isolation of haploid *C. albicans* and subsequent construction of tool strains^30,31^ have opened up opportunities for performing genetic screens that had been very difficult to do in the diploids. We have constructed several small gene-deletion libraries based on gene functions, one of which includes genes encoding GTPases and their regulators. By screening this library, we have identified new regulators, *GYP1* and *IRA2*, of hyphal growth and biofilm formation, two traits crucial for virulence. Importantly, the findings have been validated in *C. albicans* diploids^32^, demonstrating the enormous potential of conducting large-scale genetic screens to find new genes important for virulence as well as other biological processes. In this study, we have again presented a case of successfully identifying a new regulator of the white-to-opaque switching by first screening a haploid mutant library.

## Methods

### Strains, plasmids, and growth conditions

The *C. albicans* strains and plasmids used in this study are described in Table S1 and S2, respectively. All haploid strains were verified for ploidy by flow cytometry analysis according to the published protocol^31^. Recombinant DNA manipulations were performed according to standard methods. *E. coli* XL1 blue (Stratagene) was used as the host strain for recombinant plasmids and cultured in LB broth (0.5% yeast extract, 1% tryptone, and 0.5% NaCI, pH 7.0) supplemented with 100 μg/ml ampicillin. Site-directed mutagenesis followed the manual of the Quikchange multi-site-directed mutagenesis kit (Agilent Technologies). *C. albicans* cells were routinely grown at 30 °C in YPD (2% yeast extract, 1% peptone, and 2% glucose), or GMM (glucose minimal medium, 6.79 g/l yeast nitrogen base without amino acids, and 2% glucose) supplemented with appropriate amino acids and other compounds (80 μg/ml uridine, 40 μg/ml arginine, 40 μg/ml histidine, and 1 mg/ml 5-Fluoroorotic acid) when necessary. Solid medium plates were prepared by adding 2% of agar. Lee’s medium containing N-acetylglucosamine as the carbon source (Lee’s GlcNAc) was prepared as previously described^26^ and the pH was adjusted to 6.0. Transformation of *C. albicans* with plasmids containing prototrophic markers was performed using the Fast Yeast Transformation Kit (G-Biosciences). The electroporation method^31^ was used to transform *C. albicans* with plasmids containing *SAT1* as the selection marker, and transformants were selected on YPD plates containing 200 μg/ml nourseothricin (Jena Biosciences). All gene deletions were verified by colony PCR as described^31^, and looping out of *URA3* via FLP-mediated excision followed the previous protocol^60^.

### White-to-opaque switching assays

To induce the white-to-opaque switching in haploid strains, cells were first grown on YPD plates at 25 °C for 1–2 days, then diluted with water and plated onto YPD plates (pH 6.0) containing 5 μg/ml of PB. The plates were incubated in the dark at 25 °C for 7 days to allow the development of single colonies (around 50–80 colonies per plate). The plates were then photographed with a digital camera and the numbers of colonies exhibiting different color patterns were counted. Cells from representative colonies were picked and suspended into water for morphological examination under an optical microscope (Leica LEITZ DM RZ) equipped with a Moticam 10 digital camera. Images were acquired with the Motic Images Plus 2.0 ML software.

To induce the white-to-opaque switch in diploid strains, cells were first grown on GMM plates at 25 °C for 3–4 days, then diluted with water and plated onto Lee’s GlcNAc plates (pH 6.0) containing 5 μg/ml of PB. The plates were incubated in the dark at 25 °C for 6 days to allow the development of single colonies (around 100–150 colonies per plate) and then photographed. The colonies exhibiting different color patterns were counted. Switching frequency was calculated as the percentage of red colonies and white colonies with red sectors over the number of total colonies. Cells from representative colonies were picked and suspended into water for morphological examination under Leica DMRXA2 microscope equipped with a Coolsnap HQ2 digital camera (Roper Scientific). Differential interference contrast (DIC) images were acquired using the MetaMorph 7.5 software (MDS Analytical Technologies).

### Imaging of dTomato signals

Haploid strains transformed with dTomato-expressing plasmid were first grown on YPD plates at 25 °C for 1–2 days, then diluted with water and plated onto YPD plates (pH 6.0) without PB. The plates were incubated in the dark at 25 °C for 7 days to allow the development of single colonies (around 50–80 colonies per plate). The plates were then examined under Olympus MVX10 fluorescent microscope equipped with Olympus DP71 digital camera through transmitted bright field (TBF) and RFP channels to visualize the dTomato signal on the colony level. Images were acquired with the DP Controller software and the number of colonies with or without the dTomato signal were counted. Cells from representative colonies were also picked and suspended into water for morphological examination under a florescent microscope (Leica DM RXA2). Images were acquired using the MetaMorph 7.5 software through DIC and RFP filters to visualize the dTomato signal on the cellular level.

### Protein extraction, immunoprecipitation (IP), and Western blotting (WB)

To prepare yeast lysates, cells were harvested into 2 ml screw-cap microcentrifuge tubes by brief centrifugation to obtain pellets with a volume ≤ 500 μl and resuspended in 500 μl of ice-cold yeast lysis buffer (50 mM Tris-HCl [pH 7.4], 150 mM KCl, 1% NP-40) containing the protease inhibitor cocktail (Nacalai Tesque Inc). After adding an equal volume of acid-washed glass beads (Sigma-Aldrich), cells were broken by 6 rounds of 60-second beating at 5000 rpm in a MicroSmash MS-100 beater (TOMY Medico) with 1 min of cooling on ice between rounds. The lysed cells were then centrifuged at 16,000 rpm for 15 min at 4 °C and supernatants were collected.

To perform IP, yeast lysate was incubated with 30 μl of the slurry of rabbit polyclonal HA or Myc beads (Santa Cruz Biotechnology) at 4 °C for 1 h. After brief centrifugation at 800 rpm, the beads were washed 3 times by resuspension in 1 ml cold yeast lysis buffer followed by brief centrifugation. The washed beads were resuspended in 20 μl 2x protein loading buffer (125 mM Tris-HCl [pH 6.8], 4% SDS, 20% glycerol, 200 mM dithiothreitol, 0.02% bromophenol blue) and boiled for 10 min. Protein samples were separated by 10% SDS-PAGE gel and subsequently transferred to a PVDF membrane (Bio-Rad Laboratories).

For WB analysis, the PVDF membrane was first incubated with 5% milk blocking solution (dissolved in PBS containing 0.1% Tween-20, PBST) at room temperature for 1 h or at 4 °C overnight. After a brief rinse with PBST, the membrane was incubated with PBST containing a 1:1000 diluted primary antibody (mouse monoclonal HA or mouse monoclonal Myc antibody from Nacalai Tesque Inc) at room temperature for 1 h, followed by 3 rounds of 5-min wash with PBST. The membrane was then incubated with PBST containing a 1:5000 diluted secondary antibody (HRP-linked anti-mouse IgG from sheep; GE Healthcare). After 3 rounds of 5-min wash with PBST, the membrane was immersed in Pierce ECL WB substrate solution (Thermo Scientific) and exposed to X-film.

### Statistical analysis

The white-to-opaque switching frequency was calculated as the percentage (with standard deviation) of red/pink-containing colonies (red/pink colonies and white colonies with red/pink regions) over total colonies. Statistical analyses were performed using two-tailed, unpaired Student’s *t*-tests in Excel. p values < 0.05 were considered significant. All experiments were carried out at least in triplicate.

## Electronic supplementary material

Supplementary Information

## References

[1] Calderone R, Clancy C (2012). Candida and candidiasis.

[2] Brown GD (2012). Hidden killers: human fungal infections. Sci. Transl. Med..

[3] Jacobsen ID (2012). *Candida albicans* dimorphism as a therapeutic target. Expert Rev. Anti. Infect. Ther..

[4] Soll, D. R. The role of phenotypic switching in the basic biology and pathogenesis of *Candida albicans*. J. Oral Microbiol. **6**, 10.3402/jom.v6.22993 (2014). 10.3402/jom.v6.22993 PMC3895265 24455104

[5] Slutsky B (1987). “White-opaque transition”: a second high-frequency switching system in *Candida albicans*. J. Bacteriol..

[6] Anderson JM, Soll DR (1987). Unique phenotype of opaque cells in the white-opaque transition of *Candida albicans*. J. Bacteriol..

[7] Anderson J, Mihalik R, Soll DR (1990). Ultrastructure and antigenicity of the unique cell wall pimple of the *Candida* opaque phenotype. J. Bacteriol..

[8] Lockhart SR (2002). *Candida albicans*, white-opaque switchers are homozygous for mating type. Genetics.

[9] Miller MG, Johnson AD (2002). White-opaque switching in *Candida albicans* is controlled by mating-type locus homeodomain proteins and allows efficient mating. Cell.

[10] Bennett RJ (2015). The parasexual lifestyle of *Candida albicans*. Curr. Opin. Microbiol..

[11] Daniels KJ, Srikantha T, Lockhart SR, Pujol C, Soll DR (2006). Opaque cells signal white cells to form biofilms in *Candida albican*s. EMBO J..

[12] Park YN, Daniels KJ, Pujol C, Srikantha T, Soll DR (2013). *Candida albicans* forms a specialized “sexual” as well as “pathogenic” biofilm. Eukaryot. Cell.

[13] Geiger J, Wessels D, Lockhart SR, Soll DR (2004). Release of a potent polymorphonuclear leukocyte chemoattractant is regulated by white-opaque switching in *Candida albicans*. Infect. Immun..

[14] Sasse C, Hasenberg M, Weyler M, Gunzer M, Morschhauser J (2013). White-opaque switching of *Candida albicans* allows immune evasion in an environment-dependent fashion. Eukaryot. Cell.

[15] Huang G (2006). Bistable expression of *WOR1*, a master regulator of white-opaque switching in *Candida albicans*. Proc. Natl. Acad. Sci. USA.

[16] Srikantha T (2006). *TOS9* regulates white-opaque switching in *Candida albicans*. Eukaryot. Cell.

[17] Zordan RE, Galgoczy DJ, Johnson AD (2006). Epigenetic properties of white-opaque switching in *Candida albicans* are based on a self-sustaining transcriptional feedback loop. Proc. Natl. Acad. Sci. USA.

[18] Zordan RE, Miller MG, Galgoczy DJ, Tuch BB, Johnson AD (2007). Interlocking transcriptional feedback loops control white-opaque switching in *Candida albicans*. PLoS biology.

[19] Hernday AD (2013). Structure of the transcriptional network controlling white-opaque switching in *Candida albicans*. Mol. Microbiol..

[20] Tuch BB (2010). The transcriptomes of two heritable cell types illuminate the circuit governing their differentiation. PLoS Genet..

[21] Srikantha T, Tsai L, Daniels K, Klar AJ, Soll DR (2001). The histone deacetylase genes HDA1 and RPD3 play distinct roles in regulation of high-frequency phenotypic switching in *Candida albicans*. J. Bacteriol..

[22] Hnisz D, Schwarzmuller T, Kuchler K (2009). Transcriptional loops meet chromatin: a dual-layer network controls white-opaque switching in *Candida albicans*. Mol. Microbiol..

[23] Stevenson JS, Liu H (2011). Regulation of white and opaque cell-type formation in *Candida albicans* by Rtt109 and Hst3. Mol. Microbiol..

[24] Huang G, Srikantha T, Sahni N, Yi S, Soll DR (2009). CO(2) regulates white-to-opaque switching in *Candida albicans*. Curr. Biol..

[25] Sun Y (2015). pH Regulates White-Opaque Switching and Sexual Mating in *Candida albicans*. Eukaryot. Cell.

[26] Huang G (2010). N-acetylglucosamine induces white to opaque switching, a mating prerequisite in *Candida albicans*. PLoS Pathog..

[27] Morrow B, Anderson J, Wilson J, Soll DR (1989). Bidirectional stimulation of the white-opaque transition of *Candida albicans* by ultraviolet irradiation. J. Gen. Microbiol..

[28] Alby K, Bennett RJ (2009). Stress-induced phenotypic switching in *Candida albicans*. Mol. Biol. Cell..

[29] Zou H, Fang HM, Zhu Y, Wang Y (2010). *Candida albicans* Cyr1, Cap1 and G-actin form a sensor/effector apparatus for activating cAMP synthesis in hyphal growth. Mol. Microbiol..

[30] Hickman MA (2013). The ‘obligate diploid’ *Candida albicans* forms mating-competent haploids. Nature.

[31] Zeng G, Wang YM, Chan FY, Wang Y (2014). One-step targeted gene deletion in *Candida albicans* haploids. Nat. Protoc..

[32] Seneviratne CJ (2015). New “haploid biofilm model” unravels *IRA2* as a novel regulator of *Candida albicans* biofilm formation. Sci. Rep..

[33] Huang, Z. X., Wang, H., Wang, Y. M. & Wang, Y. Novel mechanism coupling cyclic AMP-protein kinase A signaling and golgi trafficking via Gyp1 phosphorylation in polarized growth. *Eukaryot. Cell* **13** (2014). 10.1128/EC.00231-14 PMC4248690 25326521

[34] Jaffe AB, Hall A (2005). Rho GTPases: biochemistry and biology. Annu. Rev. Cell. Dev. Biol..

[35] Schmidt A, Hall A (2002). Guanine nucleotide exchange factors for Rho GTPases: turning on the switch. Genes Dev..

[36] Moon SY, Zheng Y (2003). Rho GTPase-activating proteins in cell regulation. Trends Cell. Biol..

[37] Zheng XD, Lee RT, Wang YM, Lin QS, Wang Y (2007). Phosphorylation of Rga2, a Cdc42 GAP, by CDK/Hgc1 is crucial for *Candida albicans* hyphal growth. EMBO J..

[38] Corvest V, Bogliolo S, Follette P, Arkowitz RA, Bassilana M (2013). Spatiotemporal regulation of Rho1 and Cdc42 activity during Candida albicans filamentous growth. Mol. Microbiol..

[39] Gratacap RL, Rawls JF, Wheeler RT (2013). Mucosal candidiasis elicits NF-kappaB activation, proinflammatory gene expression and localized neutrophilia in zebrafish. Dis. Model Mech..

[40] Morrow B, Srikantha T, Anderson J, Soll DR (1993). Coordinate regulation of two opaque-phase-specific genes during white-opaque switching in *Candida albicans*. Infect. Immun..

[41] Strauss A, Michel S, Morschhauser J (2001). Analysis of phase-specific gene expression at the single-cell level in the white-opaque switching system of *Candida albicans*. J. Bacteriol..

[42] Ziman M, O’Brien JM, Ouellette LA, Church WR, Johnson DI (1991). Mutational analysis of CDC42Sc, a *Saccharomyces cerevisiae* gene that encodes a putative GTP-binding protein involved in the control of cell polarity. Mol. Cell. Biol..

[43] Dunn TM, Shortle D (1990). Null alleles of SAC7 suppress temperature-sensitive actin mutations in *Saccharomyces cerevisiae*. Mol. Cell. Biol..

[44] Schmidt A, Bickle M, Beck T, Hall MN (1997). The yeast phosphatidylinositol kinase homolog *TOR2* activates *RHO1* and *RHO2* via the exchange factor *ROM2*. Cell.

[45] Martin H, Rodriguez-Pachon JM, Ruiz C, Nombela C, Molina M (2000). Regulatory mechanisms for modulation of signaling through the cell integrity Slt2-mediated pathway in *Saccharomyces cerevisiae*. J. Biol. Chem..

[46] Green R, Lesage G, Sdicu AM, Menard P, Bussey H (2003). A synthetic analysis of the *Saccharomyces cerevisiae* stress sensor Mid2p, and identification of a Mid2p-interacting protein, Zeo1p, that modulates the PKC1-MPK1 cell integrity pathway. Microbiology.

[47] Van Aelst L, D’Souza-Schorey C (1997). Rho GTPases and signaling networks. Genes Dev..

[48] Qadota H (1996). Identification of yeast Rho1p GTPase as a regulatory subunit of 1,3-beta-glucan synthase. Science.

[49] Mazur P, Baginsky W (1996). *In vitro* activity of 1,3-beta-D-glucan synthase requires the GTP-binding protein Rho1. J. Biol. Chem..

[50] Drgonova J (1996). Rho1p, a yeast protein at the interface between cell polarization and morphogenesis. Science.

[51] Nonaka H (1995). A downstream target of RHO1 small GTP-binding protein is PKC1, a homolog of protein kinase C, which leads to activation of the MAP kinase cascade in *Saccharomyces cerevisiae*. EMBO J..

[52] Kamada Y (1996). Activation of yeast protein kinase C by Rho1 GTPase. J. Biol. Chem..

[53] Hirano H (1996). ROM7/BEM4 encodes a novel protein that interacts with the Rho1p small GTP-binding protein in *Saccharomyces cerevisiae*. Mol. Cell. Biol..

[54] Kohno H (1996). Bni1p implicated in cytoskeletal control is a putative target of Rho1p small GTP binding protein in *Saccharomyces cerevisiae*. EMBO J..

[55] Guo W, Tamanoi F, Novick P (2001). Spatial regulation of the exocyst complex by Rho1 GTPase. Nat. Cell Biol..

[56] Kondoh O, Tachibana Y, Ohya Y, Arisawa M, Watanabe T (1997). Cloning of the RHO1 gene from *Candida albicans* and its regulation of beta-1,3-glucan synthesis. J. Bacteriol..

[57] Smith SE, Csank C, Reyes G, Ghannoum MA, Berlin V (2002). *Candida albicans RHO1* is required for cell viability *in vitro* and *in vivo*. FEMS Yeast Res..

[58] Xie JL (2016). Signaling through Lrg1, Rho1 and Pkc1 Governs Candida albicans Morphogenesis in Response to Diverse Cues. PLoS Genet..

[59] Levin DE, Errede B (1995). The proliferation of MAP kinase signaling pathways in yeast. Curr. Opin. Cell Biol..

[60] Morschhauser J, Michel S, Staib P (1999). Sequential gene disruption in *Candida albicans* by FLP-mediated site-specific recombination. Mol. Microbiol..

