# Supplementary material for: Sac7 and Rho1 regulate the white-to-opaque switching in Candida albicans
Source: Sci Rep. 2018 Jan 17;8:875. doi: 10.1038/s41598-018-19246-9 (PMC5772354; doi:10.1038/s41598-018-19246-9)
Supplement: Supplementary file 1 — Supplementary Information [file 41598_2018_19246_MOESM1_ESM.pdf]

Supplementary Information

Sac7 and Rho1 regulate the white-to-opaque switching in *Candida albicans*

Siwy Ling Yang[1¶], Guisheng Zeng[1¶], Fong Yee Chan[1], Yan-Ming Wang[1], Dongliang Yang[2], and Yue Wang[1, 3]*

[1]Institute of Molecular and Cell Biology, 61 Biopolis Drive, Proteos, Singapore 138673; [2]Institute of Advanced Materials, Nanjing University of Posts and Telecommunications, Nanjing, Jiangsu 210046, People's Republic of China; [3]Department of Biochemistry, Yong Loo Lin School of Medicine, National University of Singapore, Singapore 117597

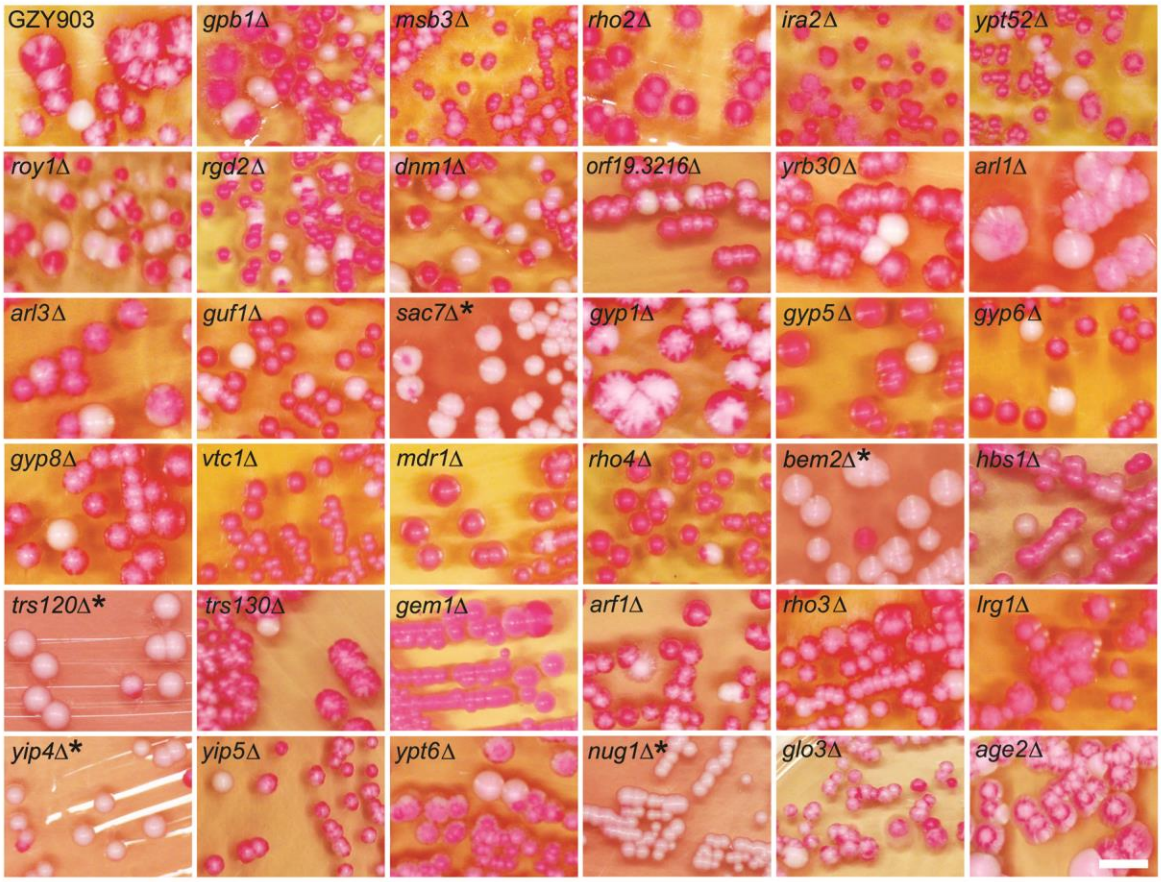

Fig. S1: Wild-type (GZY903) and haploid deletion mutants (as indicated) were streaked onto YPD (pH 6.0, +PB) plates and incubated at 30ºC for 7 days to allow the formation of single colonies. Representative images of colonies from each strain are shown. Asterisks indicate mutants with much less red colonies than **the wild type.** Bar, 5 mm.

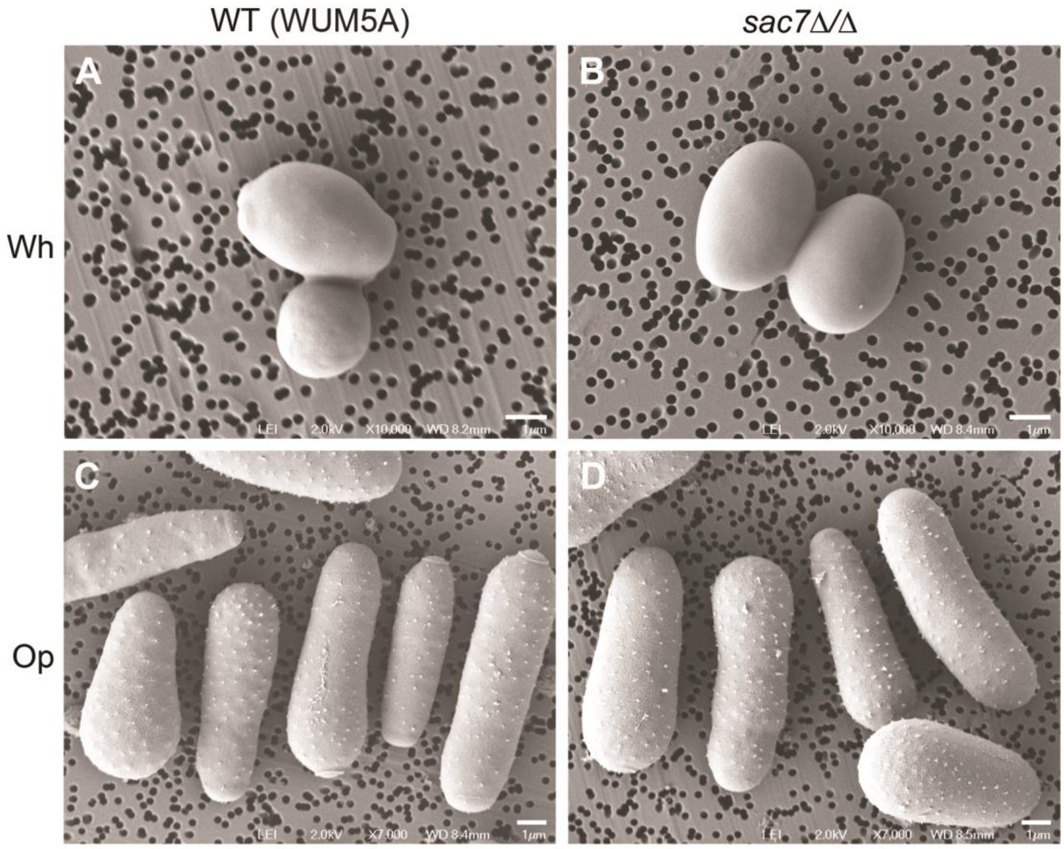

Fig. S2: Scanning electron micrographs of white (**A** and **B**) and opaque (**C** and **D**) cells. WT (WUM5A, A and C) and *sac7*Δ/Δ (YSL504, B and D) cells were grown on Lee's GlcNAc plates (pH 6.0, +PB) in the dark at 25ºC for 6 days. Cells from white and red colonies of each strain were then grown in liquid Lee's medium with 1.25% glucose (white) or with 1.25% GlcNAc (Red) at 25ºC for 3 days. To perform SEM, cells were harvested and washed once with 0.1M sodium cacodylate buffer (pH 7.4).Cells were then resuspended in fixation buffer (2.5% glutaradehyde in 0.1M sodium cacodylate buffer, pH 7.4) and incubated on shaker for 30 min at 25ºC. Post-staining was done in 1% osmium tetraoxide before gradual dehydration in increasing ethanol concentrations. Dehydrated cells were dried in a critical point dryer before being mounted and sputter coated with gold (Leica). Samples were viewed in a Jeol 6701F field emission SEM.

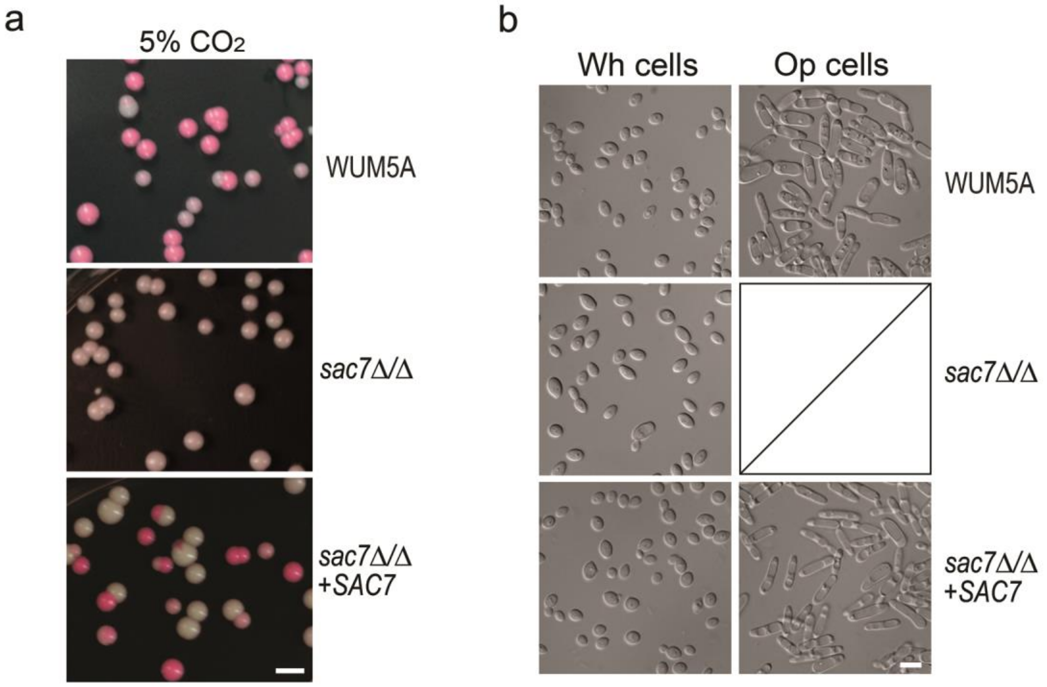

c

Strain	Wh colonies	Op colonies or wh colonies with op regions	Total colonies	Switching frequencies (%)
WUM5A	105	291	396	73.4 ± 2.4
sac7Δ/Δ	721	0	721	0.0 ± 0.0
sac7Δ/Δ+SAC7	174	149	323	46.4 ± 4.4

Fig. S3: The *sac7Δ/Δ* mutant is defective in CO_2-induced white-to-opaque switching. (**a**) Cells of WUM5A (WT), *sac7Δ/Δ* (YSL504), and *sac7Δ/Δ+SAC7* (YSL532) were spread onto Lee's glucose plates (pH 6.0, +PB) and incubated in the presence of 5% CO_2 at 25°C in the dark for 4 days before photography. (**b**) Cells from randomly selected red and white colonies of the tested strains were suspended in water and examined under a microscope. (**c**) The white-to-opaque switching frequency for GH1322, *sac7Δ/Δ*, and *sac7Δ/Δ+SAC7* tested on Lee's glucose plates (pH 6.0,+PB) in the presence of 5% CO_2. *, $p<0.05$ (Student's *t*-test).

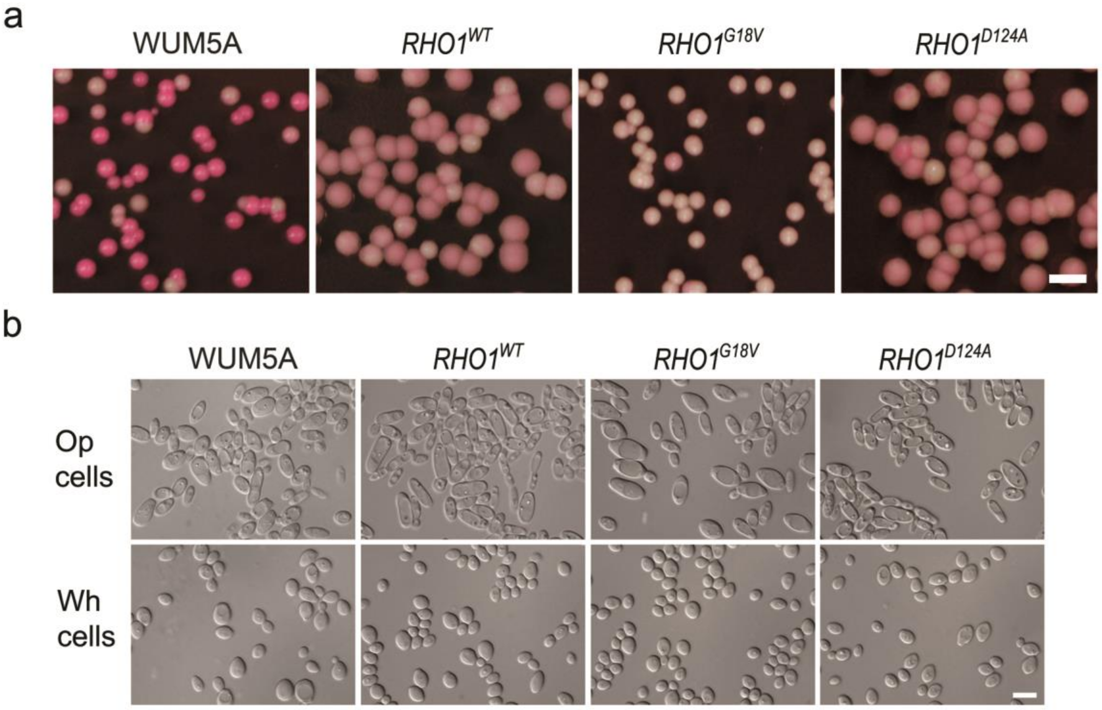

c

Strain	Wh colonies	Op colonies or wh colonies with op regions	Total colonies	Switching frequencies (%)
WUM5A	132	486	618	78.6±2.0
RHO1^{WT}	204	352	556	63.3±2.2
RHO1^{G18V}	325	41	366	11.2±2.3 *
RHO1^{D124A}	63	464	527	88.0±3.2 *

Fig. S4: Rho1 negatively regulates CO_2-induced white-to-opaque switching. (**a**) Cells of WUM5A or WUM5A transformed with *RHO1*^{WT} (YSL617), *RHO1*^{G18V} (YSL608), or *RHO1*^{D124A} (YSL613) were grown on Lee's glucose plates (pH6.0, +PB) in the presence of 5% CO_2 at 25°C in the dark for 4 days. Colonies formed by each strain were photographed and representative images are shown. (**b**) Representative images of white and opaque cells from the indicated strains. (**c**) CO_2-induced white-to-opaque switching frequency for WUM5A, WUM5A transformed with *RHO1*^{WT}, *RHO1*^{G18V}, or *RHO1*^{D124A} strains. *, $p<0.05$ (Student's *t*-test).

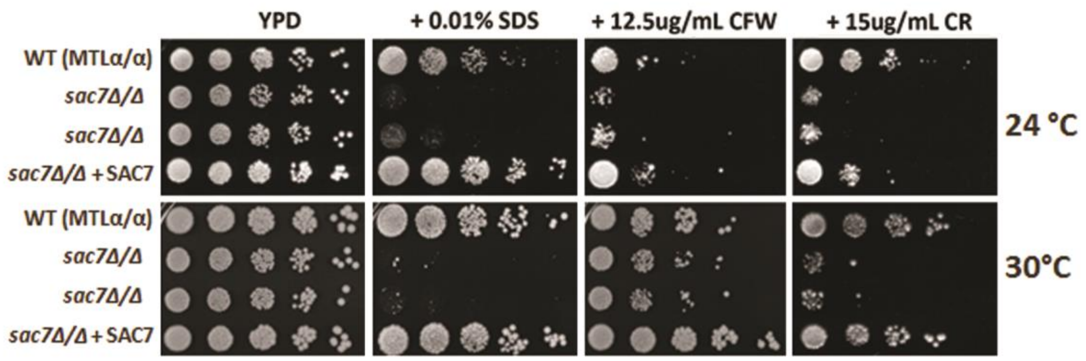

Fig. S5: Wild-type (WUM5A), *sac7*Δ/Δ (YSL504) and *sac7*Δ/Δ+*SAC7* (YSL532) were grown in YPD at 30℃ overnight. The cultures were then adjusted to OD600=1, and subjected to 10 time dilution, followed by 5 times serial dilutions. 3 ul of the diluted cultures were spotted on plates of YPD, YPD with 0.01% SDS, YPD with12.5 μg/ml calcofluor white (CFW), YPD with 15 μg/ml congo red (CR), and incubated at 25°C and 30°C for 2-3 days.

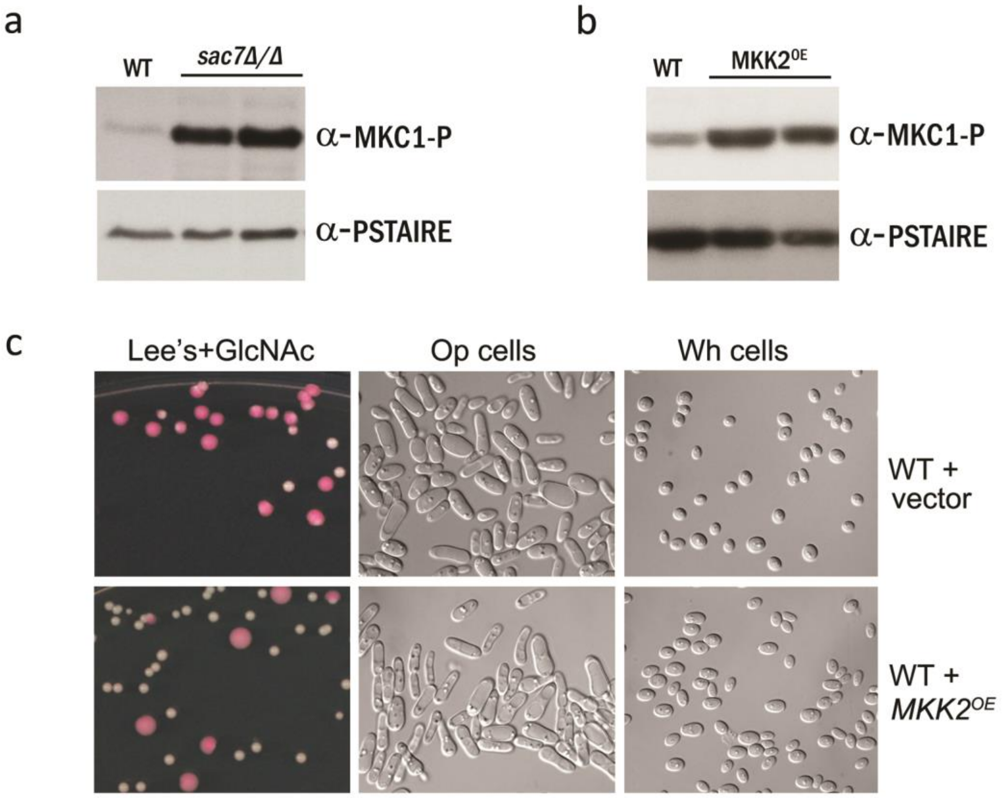

d

Strain	Wh colonies	Op colonies or Wh colonies with Op regions	Total colonies	Switching frequencies (%)
WT + vector	48	254	302	84.1 ± 4.7] *
WT + *MKK2*^{OE}	335	64	399	16.0 ± 5.4]

Fig. S6: (**a** and **b**) Increase of Mkc1 phosphorylation level in *sac7*Δ/Δ (a) and *MKK2* overexpression (b) cells. Protein extracts were prepared from WT (WUM5A), *sac7*Δ/Δ (YSL504), and *MKK2* overexpression (YSL643) cells and subjected to Western analysis with anti-phosphorylated-Mkc1 antibody (Cell Signaling). The same membrane was striped and probed with anti-Cdc28 antibody (α-PSTAIRE, Santa Cruz) to serve as loading control. (**c**) WT cells transformed with empty vector (pYGS860) or *MKK2* overexpression plasmid (pYSL93) were spread onto Lee's GlcNAc (pH 6, +PB) and incubated at 25ºC in dark for 6 days before photography. Cells from red and white colonies of each strain were suspended into water and examined under a microscope for image acquisition. (**d**) White and red colonies (including white colonies with red regions) of each strain were counted for the calculation of white-to-opaque switching frequency. *, $p<0.05$ (Student's *t*-test).

Supplementary Table 1. Yeast strains used in this study

Strain	Genotype/Description	Source/Parent
GZY803	*MTLα his4 ura3Δ::HIS4*	(1)
GZY885	(*gpb1Δ*) *MTLα his4 ura3Δ::HIS4 gpb1Δ::UFP*; deletion of *GPB1* in GZY803 by using pYGS1084	GZY803
GZY886	(*msb3Δ*) *MTLα his4 ura3Δ::HIS4 msb3Δ::UFP*; deletion of *MSB3* in GZY803 by using pYGS1087	GZY803
GZY887	(*rho2Δ*) *MTLα his4 ura3Δ::HIS4 rho2Δ::UFP*; deletion of *RHO2* in GZY803 by using pYGS1086	GZY803
GZY893	(*ira2Δ*) *MTLα his4 ura3Δ::HIS4 ira2Δ::UFP*; deletion of *IRA2* in GZY803 by using pYGS1089	GZY803
GZY895	(*ypt52Δ*) *MTLα his4 ura3Δ::HIS4 ypt52Δ::UFP*; deletion of *YPT52* in GZY803 by using pYGS1091	GZY803
GZY897	(*roy1Δ*) *MTLα his4 ura3Δ::HIS4 roy1Δ::UFP*; deletion of *ROY1* in GZY803 by using pYGS1093	GZY803
GZY898	(*rgd2Δ*) *MTLα his4 ura3Δ::HIS4 rgd2Δ::UFP*; deletion of *RGD2* in GZY803 by using pYGS1090	GZY803
GZY903	(GZY803+*URA3*) *MTLα his4 ura3Δ::HIS4 URA3*; integration of pYGS860 into GZY803	GZY803
GZY904	(*dmn1Δ*) *MTLα his4 ura3Δ::HIS4 dmn1Δ::UFP*; deletion of *DMN1* in GZY803 by using pYGS1100	GZY803
GZY905	(*orf19.3216Δ*) *MTLα his4 ura3Δ::HIS4 orf19.3216Δ::UFP*; deletion of *ORF19.3216* in GZY803 by using pYGS1103	GZY803
GZY908	(*yrb30Δ*) *MTLα his4 ura3Δ::HIS4 yrb30Δ::UFP*; deletion of *YRB30* in GZY803 by using pYGS1104	GZY803
GZY912	(*arl1Δ*) *MTLα his4 ura3Δ::HIS4 arl1Δ::UFP*; deletion of *ARL1* in GZY803 by using pYGS1108	GZY803
GZY913	(*arl3Δ*) *MTLα his4 ura3Δ::HIS4 arl3Δ::UFP*; deletion of *ARL3* in GZY803 by using pYGS1109	GZY803
GZY915	(*guf1Δ*) *MTLα his4 ura3Δ::HIS4 guf1Δ::UFP*; deletion of *GUF1* in GZY803 by using pYGS1112	GZY803
GZY916	(*sac7Δ*) *MTLα his4 ura3Δ::HIS4 sac7Δ::UFP*; deletion of *SAC7* in GZY803 by using pYGS1088	GZY803
GZY950	(*sac7Δ*) *MTLα his4 ura3Δ::HIS4 sac7Δ::FRT*; loop out of *URA3* from GZY916	GZY916
GZY1092	(*sac7Δ+URA3*) *MTLα his4 ura3Δ::HIS4 sac7Δ::FRT URA3*; integration of pYGS860 into GZY950	GZY950
GZY1121	(GZY803+*URA3+dTMT*) *MTLα his4 ura3Δ::HIS4 URA3 OP4::dTomoto-SAT1*; integration of pYSL54 into GZY903	GZY903
GZY1141	(*sac7Δ+URA3+dTMT*) *MTLα his4 ura3Δ::HIS4 sac7Δ::FRT URA3 OP4::dTomoto-SAT1*; integration of pYSL54 into GZY1092	GZY1092
GZY1146	(*sac7Δ+SAC7*) *MTLα his4 ura3Δ::HIS4 sac7Δ::FRT sac7::SAC7-URA3*; integration of pYSL60 into GZY950	GZY950
GZY1153	(*sac7Δ+SAC7+dTMT*) *MTLα his4 ura3Δ::HIS4 sac7Δ::FRT sac7::SAC7-URA3 OP4::dTomoto-SAT1*; integration of pYSL54 into GZY1146	GZY1146
GZY1130	(*sac7Δ+WOR1*^{OE}*+dTMT*) *MTLα his4 ura3Δ::HIS4 sac7Δ::FRT wor1::TetO-Myc-WOR1-TetR-URA3 OP4::dTomoto-SAT1*; integration of pYSL54 into YSL36	YSL36
YGJ01	(*yip4Δ*) *MTLα his4 ura3Δ::HIS4 yip4Δ::UFP*; deletion of *YIP4* in GZY803 by using pGJX01	GZY803
YGJ02	(*yip5Δ*) *MTLα his4 ura3Δ::HIS4 yip5Δ::UFP*; deletion of *YIP5* in GZY803 by using pGJX02	GZY803
YGJ03	(*ypt6Δ*) *MTLα his4 ura3Δ::HIS4 ypt6Δ::UFP*; deletion of *YPT6* in GZY803 by using pGJX03	GZY803
YGJ04	(*nug1Δ*) *MTLα his4 ura3Δ::HIS4 nug1Δ::UFP*; deletion of *NUG1* in GZY803 by using pGJX04	GZY803
YGJ05	(*glo3Δ*) *MTLα his4 ura3Δ::HIS4 glo3Δ::UFP*; deletion of *GLO3* in GZY803 by using pGJX05	GZY803
YGJ06	(*age2Δ*) *MTLα his4 ura3Δ::HIS4 age2Δ::UFP*; deletion of *AGE2* in GZY803 by using pGJX06	GZY803
YHZ01	(*gyp1Δ*) *MTLα his4 ura3Δ::HIS4 gyp1Δ::UFP*; deletion of *GYP1* in GZY803 by using pHZX01	GZY803
YHZ02	(*gyp5Δ*) *MTLα his4 ura3Δ::HIS4 gyp5Δ::UFP*; deletion of *GYP5* in GZY803 by using pHZX02	GZY803

YHZ03	(*gyp6Δ*) *MTLα his4 ura3Δ::HIS4 gyp6Δ::UFP*; deletion of *GYP6* in GZY803 by using pHZX03	GZY803
YHZ04	(*gyp8Δ*) *MTLα his4 ura3Δ::HIS4 gyp8Δ::UFP*; deletion of *GYP8* in GZY803 by using pHZX04	GZY803
YWH01	(*vtc1Δ*) *MTLα his4 ura3Δ::HIS4 vtc1Δ::UFP*; deletion of *VTC1* in GZY803 by using pWHT01	GZY803
YWH02	(*mdr1Δ*) *MTLα his4 ura3Δ::HIS4 mdr1Δ::UFP*; deletion of *MDR1* in GZY803 by using pWHT02	GZY803
YWH03	(*rho4Δ*) *MTLα his4 ura3Δ::HIS4 rho4Δ::UFP*; deletion of *RHO4* in GZY803 by using pWHT03	GZY803
YWH04	(*bem2Δ*) *MTLα his4 ura3Δ::HIS4 bem2Δ::UFP*; deletion of *BEM2* in GZY803 by using pWHT04	GZY803
YWH05	(*hbs1Δ*) *MTLα his4 ura3Δ::HIS4 hbs1Δ::UFP*; deletion of *HBS1* in GZY803 by using pWHT05	GZY803
YWH06	(*trs120Δ*) *MTLα his4 ura3Δ::HIS4 trs120Δ::UFP*; deletion of *TRS120* in GZY803 by using pWHT06	GZY803
YWH07	(*trs130Δ*) *MTLα his4 ura3Δ::HIS4 trs130Δ::UFP*; deletion of *TRS130* in GZY803 by using pWHT07	GZY803
YWH08	(*gem1Δ*) *MTLα his4 ura3Δ::HIS4 gem1Δ::UFP*; deletion of *GEM1* in GZY803 by using pWHT08	GZY803
YWH09	(*arf1Δ*) *MTLα his4 ura3Δ::HIS4 arf1Δ::UFP*; deletion of *ARF1* in GZY803 by using pWHT09	GZY803
YWH10	(*rho3Δ*) *MTLα his4 ura3Δ::HIS4 rho3Δ::UFP*; deletion of *RHO3* in GZY803 by using pWHT10	GZY803
YWH11	(*lrg1Δ*) *MTLα his4 ura3Δ::HIS4 lrg1Δ::UFP*; deletion of *LRG1* in GZY803 by using pWHT11	GZY803
WUM5A	(GH1322) *MTLa/α ura3Δ::FRT/ura3Δ::FRT*	(2)
YSL36	(*sac7Δ+WOR1*^{OE}) *MTLα his4 ura3Δ::HIS4 sac7Δ::FRT wor1::TetO-Myc-WOR1-TetR-URA3*; integration of pYSL06 into GZY950	GZY950
YSL501	*MTLa/α ura3Δ::FRT/ura3Δ::FRT SAC7/sac7Δ::UFP;* deletion of *SAC7* in WUM5A by using pYGS1088	WUM5A
YSL502	*MTLa/α ura3Δ::FRT/ura3Δ::FRT SAC7/sac7Δ::FRT;* loop out of *URA3* from YSL501	YSL501
YSL503	(*sac7Δ/Δ*) *MTLa/α ura3Δ::FRT/ura3Δ::FRT sac7Δ::UFP/sac7Δ::FRT;* deletion of *SAC7* in YSL502 by using pYSL21	YSL502
YSL504	(*sac7Δ/Δ*) *MTLa/α ura3Δ::FRT/ura3Δ::FRT sac7Δ::FRT/sac7Δ::FRT;* loop out of *URA3* from YSL503	YSL503
YSL505	(*sac7Δ/Δ+WOR1*^{OE}) *MTLa/α ura3Δ::FRT/ura3Δ::FRT sac7Δ::FRT/sac7Δ::FRT WOR1/wor1::TetO-Myc-WOR1-TetR-URA3*; integration of pYSL06 into YSL504	YSL504
YSL509	(WUM5A+vector) ; *MTLa/α ura3Δ::FRT/ura3Δ::FRT URA3*; integration of pYGS860 into WUM5A	WUM5A
YSL512	(WUM5A+*SAC7*^{OE}) *MTLa/α ura3Δ::FRT/ura3Δ::FRT SAC7/sac7::TetO-Myc-SAC7-TetR-URA3*; integration of pYSL153 into WUM5A	WUM5A
YSL532	(*sac7Δ/Δ+SAC7*) *MTLa/α ura3Δ::FRT/ura3Δ::FRT sac7Δ::FRT/sac7Δ::SAC7-URA3*; integration of pYSL60 into YSL504	YSL504
YSL564	(*Myc-RHO1*^{D124A}) *MTLa/α ura3Δ::FRT/ura3Δ::FRT RHO1/rho1::TetO-Myc- RHO1*^{D124A}*-TetR-URA3*; integration of pYGS1279 into WUM5A	WUM5A
YSL570	(*Myc-RHO1*^{G18V}) *MTLa/α ura3Δ::FRT/ura3Δ::FRT RHO1/rho1::TetO-Myc- RHO1*^{G18V}*-TetR-URA3*; integration of pYGS1280 into WUM5A	WUM5A
YSL601	(*Myc-RHO1*^{G18V}*+SAC7-HA*) *MTLa/α ura3Δ::FRT/ura3Δ::FRT RHO1/rho1::TetO-Myc- RHO1*^{G18V}*-TetR-URA3 SAC7/sac7::SAC7-HA-SAT1*; integration of pYSL79 into YSL570	YSL570
YSL607	(*SAC7-HA*) *MTLa/α ura3Δ::FRT/ura3Δ::FRT SAC7/sac7::SAC7-HA*-SAT1; integration of pYSL79 into WUM5A	WUM5A
YSL608	(*RHO1*^{G18V}) *MTLa/α ura3Δ::FRT/ura3Δ::FRT RHO1/rho1::Myc-RHO1*^{G18V}*-URA3*; integration of pYSL82 into WUM5A	WUM5A
YSL609	(*Myc-RHO1*^{D124A}*+SAC7-HA*) *MTLa/α ura3Δ::FRT/ura3Δ::FRT RHO1/rho1::TetO-Myc- RHO1*^{D124A}*-TetR-URA3 SAC7/sac7::SAC7-HA-SAT1*; integration of pYSL79 into YSL564	YSL564

YSL613	(*RHO1*^{D124A}) *MTL*a/α *ura3Δ::FRT/ura3Δ::FRT RHO1/rho1::Myc-RHO1*^{D124A}*-URA3*; integration of pYSL84 into WUM5A	WUM5A
YSL616	(*RHO1*^{G18V}+*WOR1*^{OE}) *MTL*a/α *ura3Δ::FRT/ura3Δ::FRT RHO1/rho1::Myc-RHO1*^{G18V}*-URA3 WOR1/wor1::TetO-Myc-WOR1-TetR-SAT1*; integration of pYGS1312 into YSL608	YSL608
YSL617	(*RHO1*^{WT}) *MTL*a/α *ura3Δ::FRT/ura3Δ::FRT RHO1/rho1::Myc-RHO1-URA3*; integration of pYGS1315 into WUM5A	WUM5A
YSL619	*MTL*a/α *ura3Δ::FRT/ura3Δ::FRT RAS1/ras1Δ::UFP sac7Δ::FRT/sac7Δ::FRT*; deletion of *RAS1* in YSL504 by using pYGS1317	YSL504
YSL621	*MTL*a/α *ura3Δ::FRT/ura3Δ::FRT RAS1/ras1Δ::FRT sac7Δ::FRT/sac7Δ::FRT*; loop out of *URA3* from YSL619	YSL619
YSL623	(ras1*Δ/Δ sac7Δ/Δ*) *MTL*a/α *ura3Δ::FRT/ura3Δ::FRT ras1Δ ::UFP/ras1Δ::FRT sac7Δ::FRT/sac7Δ::FRT,* deletion of *RAS1* in YSL621 by using pYGS1318	YSL621
YSL634	*MTL*a/α *ura3Δ::FRT/ura3Δ::FRT RAS1/ras1Δ::UFP*; deletion of *RAS1* in WUM5A by using pYGS1317	WUM5A
YSL637	*MTL*a/α *ura3Δ::FRT/ura3Δ::FRT RAS1/ras1Δ::FRT*; loop out of *URA3* from YSL634	YSL634
YSL639	(*ras1Δ/Δ*) *MTL*a/α *ura3Δ::FRT/ura3Δ::FRT ras1Δ::UFP/ras1Δ::FRT;* deletion of *RAS1* in GH1322 by using pYGS1318	YSL637
YSL643	WUM5A+*MKK2*^{OE}; *MTL*a/α *ura3Δ::FRT/ura3Δ::FRT MEK1/mek1::TetO-Myc-MEK1-TetR-URA3*; integration of pYSL93 into WUM5A	WUM5A

1. Hickman, M.A., Zeng, G., Forche, A., Hirakawa, M.P., Abbey, D., Harrison, B.D., Wang, Y.M., Su, C.H., Bennett, R.J., Wang, Y., Berman, J. The 'obligate diploid' *Candida albicans* forms mating competent haploids. *Nature*. 7: 55-9 (2013).
2. Strauss, A., Michel, S., Morschhauser, J. Analysis of phase-specific gene expression at the single-cell level in the white-opaque switching system of *Candida albicans*. *J Bacteriol*. 183: 3761-3769 (2001).

Supplementary Table 2. Plasmid constructs used in this study

Construct	Description
CIP10U	*C. albicans* integration vector with *URA3* as the selection marker; generated by replacing the *RP10* gene in the vector CIp10[1] with 700 bp *GAL4* untranslated region (*UTR*) at *Cla*I and *Pst*I.
pYGS860	UTR*/CIP10U; an *Asc*I site was generated at the middle of *UTR* by site-directed mutagenesis and used to linearize the plasmid for integration.
pYGS1084	GPB1Δ::UFP/pBKS; *GPB1* promoter region (~500 bp) and terminator region (~400 bp) were PCR-amplified and cloned into the vector pBKS at *Kpn*I-*Xho*I and *Not*I-*Sac*II, respectively, to flank the *URA3* flipper[2] (UFP) located between *Xho*I and *Not*I. The knock-out cassette was released by *Kpn*I and *Sac*II for transformation to generate *gpb1Δ::UFP*.
pYGS1086	RHO2Δ::UFP/pBKS; the plasmid was constructed in the same say as pYGS1084. The knock-out cassette was released by *Kpn*I and *Sac*II for transformation to generate *rho2Δ::UFP*.
pYGS1087	MSB3Δ::UFP/pBKS; the plasmid was constructed in the same say as pYGS1084. The knock-out cassette was released by *Kpn*I and *Sac*II for transformation to generate *msb3Δ::UFP*.
pYGS1088	SAC7Δ::UFP/pBKS #1; the plasmid was constructed in the same say as pYGS1084. The knock-out cassette was released by *Kpn*I and *Sac*II for transformation to generate *sac7Δ::UFP* and *sac7Δ::FRT*.
pYGS1089	IRA2Δ::UFP/pBKS; the plasmid was constructed in the same say as pYGS1084. The knock-out cassette was released by *Kpn*I and *Sac*II for transformation to generate *ira2Δ::UFP*.
pYGS1090	RGD2Δ::UFP/pBKS; the plasmid was constructed in the same say as pYGS1084. The knock-out cassette was released by *Kpn*I and *Sac*II for transformation to generate *rgd2Δ::UFP*.
pYGS1091	YPT52Δ::UFP/pBKS; the plasmid was constructed in the same say as pYGS1084. The knock-out cassette was released by *Kpn*I and *Sac*II for transformation to generate *ypt52Δ::UFP*.
pYGS1093	ROY1Δ::UFP/pBKS; the plasmid was constructed in the same say as pYGS1084. The knock-out cassette was released by *Kpn*I and *Sac*II for transformation to generate *roy1Δ::UFP*.
pYGS1100	DMN1Δ::UFP/pBKS; the plasmid was constructed in the same say as pYGS1084. The knock-out cassette was released by *Kpn*I and *Sac*II for transformation to generate *dmn1Δ::UFP*.
pYGS1103	ORF19.3216Δ::UFP/pBKS; the plasmid was constructed in the same say as pYGS1084. The knock-out cassette was released by *Kpn*I and *Sac*II for transformation to generate *orf19.3216Δ::UFP*.
pYGS1104	YRB30Δ::UFP/pBKS; the plasmid was constructed in the same say as pYGS1084. The knock-out cassette was released by *Kpn*I and *Sac*II for transformation to generate *yrb30Δ::UFP*.
pYGS1108	ARL1Δ::UFP/pBKS; the plasmid was constructed in the same say as pYGS1084. The knock-out cassette was released by *Kpn*I and *Sac*II for transformation to generate *arl1Δ::UFP*.
pYGS1109	ARL3Δ::UFP/pBKS; the plasmid was constructed in the same say as pYGS1084. The knock-out cassette was released by *Kpn*I and *Sac*II for transformation to generate *arl3Δ::UFP*.
pYGS1112	GUF1Δ::UFP/pBKS; the plasmid was constructed in the same say as pYGS1084. The knock-out cassette was released by *Kpn*I and *Sac*II for transformation to generate *guf1Δ::UFP*.
pYGS1244	TetO-Myc-RHO1^{WT}-UTR*-TetR/CIP10U; *RHO1* coding region including its terminator (1 to 1097 bp) was PCR-amplified and cloned into pYSL06 at *Cla*I-*Pst*I to replace *WOR1n*. *UTR** was then PCR-amplified and cloned at *Pac*I-*Pst*I to replace part of *RHO1* terminator. The plasmid was linearized by *Asc*I within *UTR** for integration to express *RHO1*.
pYGS1254	P_{OP4}-dTomato-UTR/CIP10U; the *C. albicans* codon-optimized *dTomato*[3] was PCR-amplified and cloned into CIP10U at *Xho*I-*Cla*I, followed by *UTR* (cloned at *Cla*I and *Pst*I). The promoter of *OP4* (-600 to -1 bp, P_{OP4}) was PCR-amplified and cloned at *Kpn*I-*Xho*I to control the expression of *dTomato*. The plasmid was linearized by *Bsa*BI within P_{OP4} for integration.
pYGS1279	TetO-Myc-RHO1^{D124A}-UTR*-TetR/CIP10U; pYGS1244 was used as the template for site-directed mutagenesis to introduce D124A mutation on RHO1. The plasmid was linearized by *Asc*I within *UTR** for integration to express *RHO1^{D124A}*.
pYGS1280	TetO-Myc-RHO1^{G18V}-UTR*-TetR/CIP10U; pYGS1244 was used as the template for site-directed mutagenesis to introduce G18V mutation on RHO1. The plasmid was linearized by *Asc*I within *UTR** for integration to express *RHO1^{G18V}*.
pYGS1312	TetO-Myc-WOR1n-TetR/CIP10SAT1; *SAT1* from pYSL54 was cloned into pYSL06 at M*lu*I-*Not*I to replace the *URA3* marker. The plasmid was linearized by *Bst*EII within *WOR1n* for integration to overexpress *WOR1* in the absence of doxycycline.

pYGS1315 Pro-Myc-RHO1^{WT}-UTR*/CIP10U; the *RHO1* promoter region (-800 to -1 bp) was PCR-amplified and cloned into CIP10U at *Kpn*I-*Xho*I to control the expression of a 6x*Myc* epitope (cloned at *Xho*I-*Cla*I). *RHO1* coding region including its terminator (1 to 842 bp) was then PCR-amplified and cloned at *Cla*I-*Pac*I to fuse in frame with the *Myc* epitope, and followed by *UTR** cloned at *Pac*I-*Pst*I. The plasmid was linearized by *Stu*I within the *RHO1* promoter region for integration.

pYGS1317 RAS1Δ::UFP/pBKS #1; the plasmid was constructed in the same say as pYGS1084. The knock-out cassette was released by *Kpn*I and *Sac*II for transformation to generate *ras1Δ::UFP* and *ras1Δ::FRT*.

pYGS1318 RAS1Δ::UFP/pBKS #2; a C-terminal region of *RAS1* (376-873 bp) was PCR-amplified and cloned into pYGS1317 at *Not*I-*Sac*II to replace the terminator region. The knock-out cassette was released by *Kpn*I and *Sac*II for transformation to generate *ras1Δ::UFP* and *ras1Δ::FRT*.

pGJX01 YIP4Δ::UFP/pBKS; the plasmid was constructed in the same say as pYGS1084. The knock-out cassette was released by *Kpn*I and *Sac*II for transformation to generate *yip4Δ::UFP*.

pGJX02 YIP5Δ::UFP/pBKS; the plasmid was constructed in the same say as pYGS1084. The knock-out cassette was released by *Kpn*I and *Sac*II for transformation to generate *yip5Δ::UFP*.

pGJX03 YPT6Δ::UFP/pBKS; the plasmid was constructed in the same say as pYGS1084. The knock-out cassette was released by *Kpn*I and *Sac*II for transformation to generate *ypt6Δ::UFP*.

pGJX04 NUG1Δ::UFP/pBKS; the plasmid was constructed in the same say as pYGS1084. The knock-out cassette was released by *Kpn*I and *Sac*II for transformation to generate *nug1Δ::UFP*.

pGJX05 GLO3Δ::UFP/pBKS; the plasmid was constructed in the same say as pYGS1084. The knock-out cassette was released by *Kpn*I and *Sac*II for transformation to generate *glo3Δ::UFP*.

pGJX06 AGE2Δ::UFP/pBKS; the plasmid was constructed in the same say as pYGS1084. The knock-out cassette was released by *Kpn*I and *Sac*II for transformation to generate *age2Δ::UFP*.

pHZX01 GYP1Δ::UFP/pBKS; the plasmid was constructed in the same say as pYGS1084. The knock-out cassette was released by *Kpn*I and *Sac*II for transformation to generate *gyp1Δ::UFP*.

pHZX02 GYP5Δ::UFP/pBKS; the plasmid was constructed in the same say as pYGS1084. The knock-out cassette was released by *Kpn*I and *Sac*II for transformation to generate *gyp5Δ::UFP*.

pHZX03 GYP6Δ::UFP/pBKS; the plasmid was constructed in the same say as pYGS1084. The knock-out cassette was released by *Kpn*I and *Sac*II for transformation to generate *gyp6Δ::UFP*.

pHZX04 GYP8Δ::UFP/pBKS; the plasmid was constructed in the same say as pYGS1084. The knock-out cassette was released by *Kpn*I and *Sac*II for transformation to generate *gyp8Δ::UFP*.

pWHT01 VTC1Δ::UFP/pBKS; the plasmid was constructed in the same say as pYGS1084. The knock-out cassette was released by *Kpn*I and *Sac*II for transformation to generate *vtc1Δ::UFP*.

pWHT02 MDR1Δ::UFP/pBKS; the plasmid was constructed in the same say as pYGS1084. The knock-out cassette was released by *Kpn*I and *Sac*II for transformation to generate *mdr1Δ::UFP*.

pWHT03 RHO4Δ::UFP/pBKS; the plasmid was constructed in the same say as pYGS1084. The knock-out cassette was released by *Kpn*I and *Sac*II for transformation to generate *rho4Δ::UFP*.

pWHT04 BEM2Δ::UFP/pBKS; the plasmid was constructed in the same say as pYGS1084. The knock-out cassette was released by *Kpn*I and *Sac*II for transformation to generate *bem2Δ::UFP*.

pWHT05 HBS1Δ::UFP/pBKS; the plasmid was constructed in the same say as pYGS1084. The knock-out cassette was released by *Kpn*I and *Sac*II for transformation to generate *hbs1Δ::UFP*.

pWHT06 TRS120Δ::UFP/pBKS; the plasmid was constructed in the same say as pYGS1084. The knock-out cassette was released by *Kpn*I and *Sac*II for transformation to generate *trs120Δ::UFP*.

pWHT07 TRS130Δ::UFP/pBKS; the plasmid was constructed in the same say as pYGS1084. The knock-out cassette was released by *Kpn*I and *Sac*II for transformation to generate *trs130Δ::UFP*.

pWHT08 GEM1Δ::UFP/pBKS; the plasmid was constructed in the same say as pYGS1084. The knock-out cassette was released by *Kpn*I and *Sac*II for transformation to generate *gem1Δ::UFP*.

pWHT09 ARF1Δ::UFP/pBKS; the plasmid was constructed in the same say as pYGS1084. The knock-out cassette was released by *Kpn*I and *Sac*II for transformation to generate *arf1Δ::UFP*.

pWHT10 RHO3Δ::UFP/pBKS; the plasmid was constructed in the same say as pYGS1084. The knock-out cassette was released by *Kpn*I and *Sac*II for transformation to generate *rho3Δ::UFP*.

pWHT11 LRG1Δ::UFP/pBKS; the plasmid was constructed in the same say as pYGS1084. The knock-out cassette was released by *Kpn*I and *Sac*II for transformation to generate *lrg1Δ::UFP*.

pYSL06	TetO-Myc-WOR1n-TetR/CIP10U; the tetracycline operator sequence (*TetO*) was cloned into CIP10U at K*pn*I-X*ho*I to control the expression of a 6x*Myc* epitope (cloned at *Xho*I-*Cla*I). The N-terminal coding region of *WOR1* (4 to 433 bp) was PCR-amplified and cloned at C*la*I-P*st*I to fuse in frame with the 6x*Myc* epitope. The tetracycline-repressible transactivator (*TetR*) was cloned after *WOR1* at *Pst*I-*Mlu*I. Both *TetO* and *TetR* were PCR-amplified from the genomic DNA of JKC1141[4]. The plasmid was linearized by *Bst*EII within *WOR1n* for integration to overexpress *WOR1* in the absence of doxycycline.
pYSL21	SAC7Δ::UFP/pBKS #2; a C-terminal region of *SAC7* (1272-1848 bp) was PCR-amplified and cloned into pYGS1088 at *Not*I-*Sac*II to replace the terminator region. The knock-out cassette was released by *Kpn*I and *Sac*II for transformation to generate *sac7Δ::UFP* and *sac7Δ::FRT*.
pYSL54	P_{OP4}-dTomato-UTR/CIP10SAT1; the nourseothricin resistance marker *SAT1* was PCR-amplified from pNIM1[5] and cloned into pYGS1254 at *Mlu*I-*Not*I to replace *URA3*. The plasmid was linearized by *Bsa*BI within P_{OP4} for integration.
pYSL60	Pro-SAC7-Ter/CIP10U ; the *SAC7* gene (including its 900 bp promoter region and 455 bp terminator region) was PCR-amplified and cloned into CIP10U between *Kpn*I and *Cla*I. The plasmid was linearized by *Bsa*BI within the promoter for integration.
pYSL79	SAC7c-HA-UTR/CIP10SAT1; the C-terminal region of *SAC7* (849 to 1845 bp) was PCR-amplified and cloned into CIP10U at *Kpn*I-*Xho*I to fuse in frame with a 2x*HA* epitope (cloned at *Xho*I-*Cla*I) and followed by *UTR* (cloned at *Cla*I-*Pst*I). *URA3* in the resulting plasmid was then replaced with *SAT1* by *Mlu*I-*Not*I digestion. The plasmid was linearized by *Nsi*I within *SAC7c* for integration.
pYSL82	Pro-Myc-$RHO1^{G18V}$-UTR*/CIP10U; pYGS1315 was used as the template for site-directed mutagenesis to introduce G18V mutation on RHO1. The plasmid was linearized by *Stu*I within the *RHO1* promoter region for integration.
pYSL84	Pro-Myc-$RHO1^{D124A}$-UTR*/CIP10U; pYGS1315 was used as the template for site-directed mutagenesis to introduce D124A mutation on RHO1. The plasmid was linearized by *Stu*I within the *RHO1* promoter region for integration.
pYSL93	TetO-Myc-MKK2-UTR*-TetR/CIP10U; *MKK2* coding region was PCR-amplified and cloned into pYGS1244 at *Cla*I-*Pac*I to replace *RHO1*. The plasmid was linearized by *Asc*I within *UTR** for integration to express *MKK2*.
pYSL153	TetO-Myc-SAC7-UTR*-TetR/CIP10U; the *SAC7* coding region (1 to 1848 bp) was PCR-amplified and cloned into pYSL06 at C*la*I-*Bst*EII to replace an N-terminal fragment of *WOR1n* and fuse in frame with the 6x*Myc* epitope. *UTR** was then PCR-amplified and cloned at *Bst*EII-*Pst*I to replace the remaining region of *WOR1n*. The plasmid was linearized by *Asc*I within *UTR** for integration to overexpress *SAC7* in the absence of doxycycline.

1. Murad, A.M., Lee, P.R., Broadbent, I.D., Barelle, C.J., and Brown, A.J. CIp10, an efficient and convenient integrating vector for *Candida albicans*. Yeast 16: 325-327 (2000).
2. Morschhauser, J., Michel, S. & Staib, P. Sequential gene disruption in *Candida albicans* by FLP-mediated site-specific recombination. *Mol Microbiol* 32: 547-556 (1999).
3. Gratacap, R.L., Rawls, J.F., Wheeler, R.T. Mucosal candidiasis elicits NF-κB activation, proinflammatory gene expression and localized neutrophilia in zebrafish. Dis Model Mech 6:1260-70 (2013).
4. Shen, J., Cowen, L.E., Griffin, A.M., Chan, L., Köhler, J.R. The *Candida albicans* pescadillo homolog is required for normal hypha-to-yeast morphogenesis and yeast proliferation. Proc Natl Acad Sci USA 105: 20918-23 (2008).Park, Y.N.,
5. Morschhauser, J. Tetracycline-Inducible Gene Expression and Gene Deletion in *Candida albicans*. Eukaryot Cell 4:1328-1342 (2005).
